# Cerebral dopamine neurotrophic factor reduces α-synuclein aggregation and propagation and alleviates behavioral alterations *in vivo*

**DOI:** 10.1016/j.ymthe.2021.04.035

**Published:** 2021-05-01

**Authors:** Katrina Albert, Diana P. Raymundo, Anne Panhelainen, Ave Eesmaa, Liana Shvachiy, Gabriela R. Araújo, Piotr Chmielarz, Xu Yan, Aastha Singh, Yraima Cordeiro, Fernando L. Palhano, Debora Foguel, Kelvin C. Luk, Andrii Domanskyi, Merja H. Voutilainen, Henri J. Huttunen, Tiago F. Outeiro, Mart Saarma, Marcius S. Almeida, Mikko Airavaara

**Affiliations:** 1 Institute of Biotechnology, HiLIFE, University of Helsinki, 00014 Helsinki, Finland; 2 Department of Brain Biochemistry, Maj Institute of Pharmacology, Polish Academy of Sciences, Krakow 31-343, Poland; 3 Neuroscience Center, HiLIFE, University of Helsinki, 00014 Helsinki, Finland; 4 Center for Neurodegenerative Disease Research, Department of Pathology and Laboratory Medicine, University of Pennsylvania, Perelman School of Medicine, Philadelphia, PA 19104, USA; 5 Herantis Pharma Plc, 20520 Espoo, Finland; 6 Instituto de Bioquímica Médica Leopoldo de Meis, Universidade Federal do Rio de Janeiro, Rio de Janeiro 21.941-902, Brazil; 7 Department of Experimental Neurodegeneration, Center for Biostructural Imaging of Neurodegeneration, University Medical Center Göttingen, 37073 Göttingen, Germany; 8 Max Planck Institute for Experimental Medicine, 37075 Göttingen, Germany; 9 Institute of Neuroscience, The Medical School, Newcastle University, Newcastle upon Tyne NE2 4HH, UK; 10 Faculdade de Farmácia, Universidade Federal do Rio de Janeiro, Rio de Janeiro 21.941-902, Brazil; 11 Protein Advanced Biochemistry, CENABIO, Universidade Federal do Rio de Janeiro, Rio de Janeiro 21.941-902, Brazil; 12 Centro Cardiovascular da Universidade de Lisboa, Faculdade de Medicina, Universidade de Lisboa, Av Prof Egas Moniz, 1649-028 Lisbon, Portugal; 13 Faculty of Pharmacy, University of Helsinki, Helsinki, Finland

**Keywords:** cerebral dopamine neurotrophic factor, synucleinopathy, CDNF, α-synuclein, Parkinson’s disease, protein aggregation, pre-formed α-synuclein fibrils, MANF, mesencephalic astrocyte-derived neurotrophic factor

## Abstract

A molecular hallmark in Parkinson’s disease (PD) pathogenesis are α-synuclein aggregates. Cerebral dopamine neurotrophic factor (CDNF) is an atypical growth factor that is mostly resident in the endoplasmic reticulum but exerts its effects both intracellularly and extracellularly. One of the beneficial effects of CDNF can be protecting neurons from the toxic effects of α-synuclein. Here, we investigated the effects of CDNF on α-synuclein aggregation *in vitro* and *in vivo*. We found that CDNF directly interacts with α-synuclein with a K_D_ = 23 ± 6 nM and reduces its auto-association. Using nuclear magnetic resonance (NMR) spectroscopy, we identified interaction sites on the CDNF protein. Remarkably, CDNF reduces the neuronal internalization of α-synuclein fibrils and induces the formation of insoluble phosphorylated α-synuclein inclusions. Intra-striatal CDNF administration alleviates motor deficits in rodents challenged with α-synuclein fibrils, though it did not reduce the number of phosphorylated α-synuclein inclusions in the *substantia nigra*. CDNF’s beneficial effects on rodent behavior appear not to be related to the number of inclusions formed in the current context, and further study of its effects on the aggregation mechanism *in vivo* are needed. Nonetheless, the interaction of CDNF with α-synuclein, modifying its aggregation, spreading, and associated behavioral alterations, provides novel insights into the potential of CDNF as a therapeutic strategy in PD and other synucleinopathies.

## Graphical abstract

**Figure:**
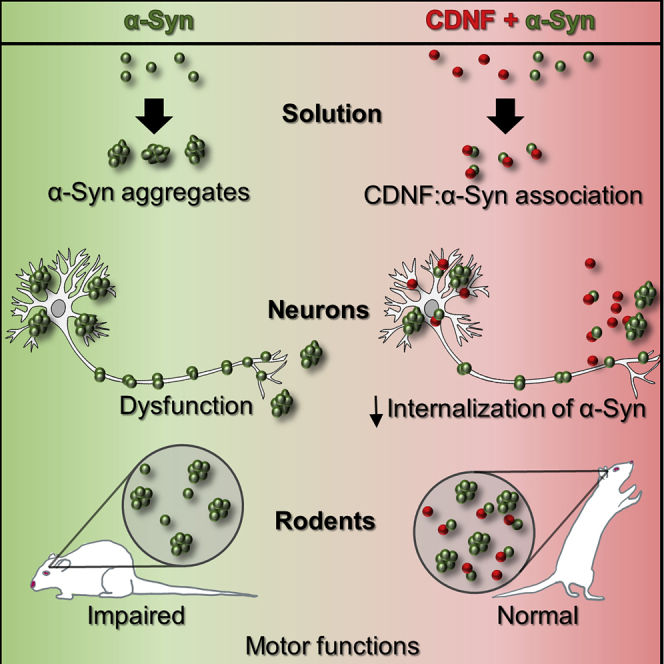

## Teaser

Airavaara, Almeida, and colleagues show that CDNF, a therapeutic protein that is in clinical trials for Parkinson’s disease, interacts with α-synuclein. α-synuclein aggregates to form Lewy bodies, often observed in postmortem brains of Parkinson’s patients. They show that this CDNF-α-synuclein interaction modifies its aggregation and may lead to functional recovery in their animal model.

## Introduction

Parkinson’s disease (PD) is a neurodegenerative disorder known for typical motor symptoms such as tremor, rigidity, and slowness of movement caused by decline in dopamine neurotransmission in the striatum, a result of loss of dopaminergic neurons in the *substantia nigra pars compacta*.^1^^,^^2^ The major pathological hallmark seen in postmortem examination of the brains of PD patients is the presence of Lewy bodies and Lewy neurites,^3^ neuronal structures rich in lipid and proteins. The major protein component of Lewy bodies is α-synuclein, which is phosphorylated at serine 129 (pSer129).^4^ Pre-formed α-synuclein fibrils (PFFs) have been developed to initiate misfolding and phosphorylation of the endogenous α-synuclein that will progressively lead to formation of pSer129 α-synuclein aggregates and Lewy body-like inclusions in the cytosol of neurons.^5^ When PFFs are stereotaxically injected into the brains of rodents, Lewy body-like inclusions are formed in particular brain structures as well as in anatomically connected areas.^6^^,^^7^ This concept of α-synuclein pathology spreading has been important in the field, since studies analyzing PD patient postmortem brains have shown similar progressive spreading of α-synuclein pathology.^8^ In line with the proposed Braak stages, it has been shown that α-synuclein can be taken up by one cell and transferred to another, although the precise mechanisms are still unknown and likely multifaceted, such as being facilitated by lysosomal membrane disruption.^9^ Cell-to-cell and neuron-to-neuron transmission of α-synuclein has nonetheless been demonstrated experimentally in *in vitro* co-culture systems using microfluidic devices showing axonal transport,^10^ in *in vivo* cell transplant studies with mouse brain,^11^ and in mice that were inoculated with α-synuclein PFFs to the gastric wall that led to Lewy body-like aggregates in the dorsal motor nucleus of the vagus nerve.^12^ In particular, the observation that dopamine neurons grafted into the brains of PD patients develop Lewy bodies over time demonstrates this phenomenon is occurring in the human brain.^13^^,^^14^

Cerebral dopamine neurotrophic factor (CDNF) and its conserved homolog mesencephalic astrocyte-derived neurotrophic factor (MANF), shown to modulate innate immunity after nerve injury,^15^^,^^16^ have neurorestorative effects when injected into the brain parenchyma in animal disease models.^17^ Their signal sequence targets CDNF into the endoplasmic reticulum (ER) lumen similarly to classical secreted neurotrophic factors. However, unlike secreted factors, CDNF and MANF have a KDEL receptor recognition sequence at the C terminus. KDEL receptors are located in the *cis*-Golgi and retrogradely transport ER retrieval sequences containing proteins to the ER lumen.^18^ These ER luminal proteins are secreted upon ER calcium depletion, and chronic ER stress contributing to neurodegeneration.^19^ After CDNF was shown to be effective in cultured cells against toxic α-synuclein oligomers,^20^ and in rodent and non-human primate PD models,^21^ the protein has entered phase I/II clinical trials in PD patients to evaluate signs of efficacy on motor functions, actigraphy measurements, and α-synuclein levels. As Lewy pathology underlies PD progression, it is of great importance to determine the effects of CDNF in α-synuclein aggregation models. In this work we implemented a variety of *in vivo* and *in vitro* α-synuclein aggregation and fibril models to study the effects of CDNF at molecular, cellular, and whole animal levels.

## Results

### CDNF reduces α-synuclein auto-association in cells

**Figure 1:**
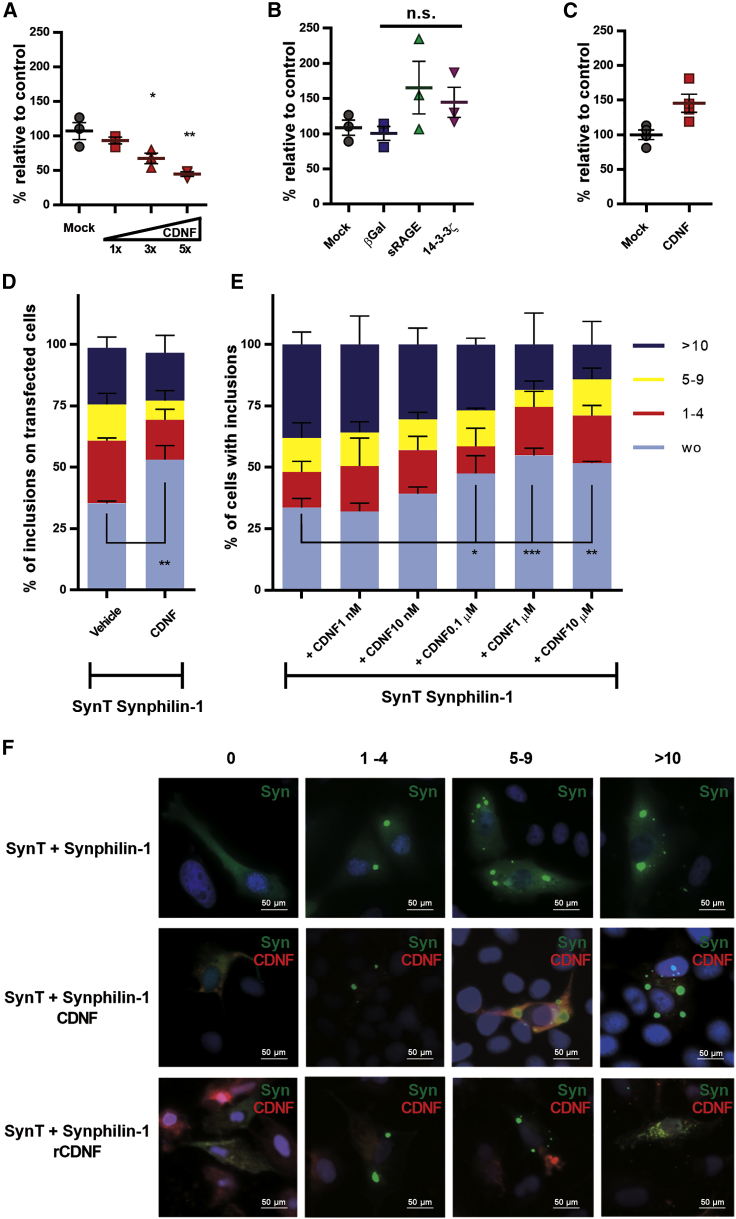
Effect of CDNF on α-synuclein auto association in cells (A–C) Neuro2A cells were transiently transfected with a pair of reporter plasmids expressing human wild-type α-synuclein fused to one of the split Gaussia luciferase (GLuc) fragments. Upon oligomerization, the interaction allows reconstitution of luciferase activity from the complementary GLuc fragments. (A) Co-expression of CDNF with its original signal sequence (mostly ER luminal protein) dose-dependently reduced α-synuclein oligomerization. Doses of CDNF expression plasmid 1× = 12.5 ng, 3× = 37.5 ng, 5× = 62.5 ng. (B) Co-expression of β-galactosidase (mainly lysosomal protein), soluble receptor for advanced glycation end products (sRAGE; a secreted protein), and 14-3-3ζ (a cytosolic protein) did not significantly alter α-synuclein oligomerization. (C) Cells co-transfected with full-length (GLuc) enzyme together with CDNF did not show decreased luciferase activity as compared to cells co-transfected with a mock plasmid. (D–F) Cells co-transfected with SynT/Synphilin-1 and CDNF with its original signal sequence, grown for 48 h (D and lines 1 and 2 in F) or pre-treated with recombinant human CDNF (E and line 3 in F) before the transient transfections with SynT/Synphilin-1. Cells were processed for immunocytochemistry with antibodies against human α-synuclein (green) or CDNF (red) and analyzed by fluorescence microscopy for the presence of inclusions. Cells were categorized according to the number of inclusions per cell: cells without inclusions (wo), between 1 to 4 (1–4), 5 to 9 (5–9), or more than 10 inclusions (>10). The scale bars represent 50 μm. The ordinary one-way ANOVA test was performed to analyze the differences between the various groups. ∗p ≤ 0.05, ∗∗p ≤ 0.01, ∗∗∗p ≤ 0.001. Error bars represent SEM.

We implemented two experimental approaches for studying the effects of CDNF on α-synuclein oligomerization in living cells. First, we used a protein-fragment complementation assay (PCA) based on the split humanized *Gaussia princeps* luciferase (GLuc) system^22^ C-terminally fused to α-synuclein. When these α-synuclein-GLuc PCA reporters are co-expressed, the bioluminescence serves as a sensitive readout of α-synuclein dimer/oligomer levels.^23^ When increasing levels of human CDNF were co-transfected to Neuro2A cells with the α-synuclein-GLuc PCA reporters, there was a notable, gene-dose-dependent reduction in α-synuclein oligomer levels (maximum reduction of 55.2% ± 3.1%; p < 0.001; Figure 1A) that was not observed with controls located to lysosomes, secretory pathway, and cytosol (Figure 1B). These data suggest that increased CDNF levels in cells specifically reduce the formation of α-synuclein oligomers. Overexpression of soluble ectodomain of receptor for advanced glycation end products (sRAGE) increased α-synuclein oligomerization to some degree, which is not statistically significant (one-way ANOVA F = 3.953, degrees of freedom [df] = 3, p = 0.0717). However, the modest increase may reflect an increased load on the ER machinery. This could be due to the fact that sRAGE has a signal peptide that targets it to the ER lumen and, together with the cytomegalovirus (CMV) promoter used here, has been shown to induce high amounts of protein expression and secretion when expressed in cells.^24^ Moreover, co-transfection of CDNF with full-length GLuc did not show decreased luciferase activity (Figure 1C).

Additionally, we used another established model based on the co-expression of a C-terminally modified form of α-synuclein (SynT) and synphilin-1, and this approach can be used to measure the intracellular α-synuclein oligomeric inclusions.^25^ We co-transfected the cells with SynT, Synphilin-1, and a plasmid encoding CDNF with its native signal peptide. The formation of intracellular inclusions was assessed 48 h post-transfection, and cells were categorized according to the number of inclusions. We observed an increase in the percentage of cells without inclusions in cells transformed with the plasmid encoding CDNF (p < 0.01; Figure 1D). In parallel, cells transfected only with SynT/Synphilin-1 were treated with increasing concentrations of recombinant CDNF protein. This treatment also increased the percentage of cells without α-synuclein inclusions and decreased the overall number of inclusions per cell (Figure 1E).

### α-synuclein and CDNF interact *in vitro*

**Figure 2:**
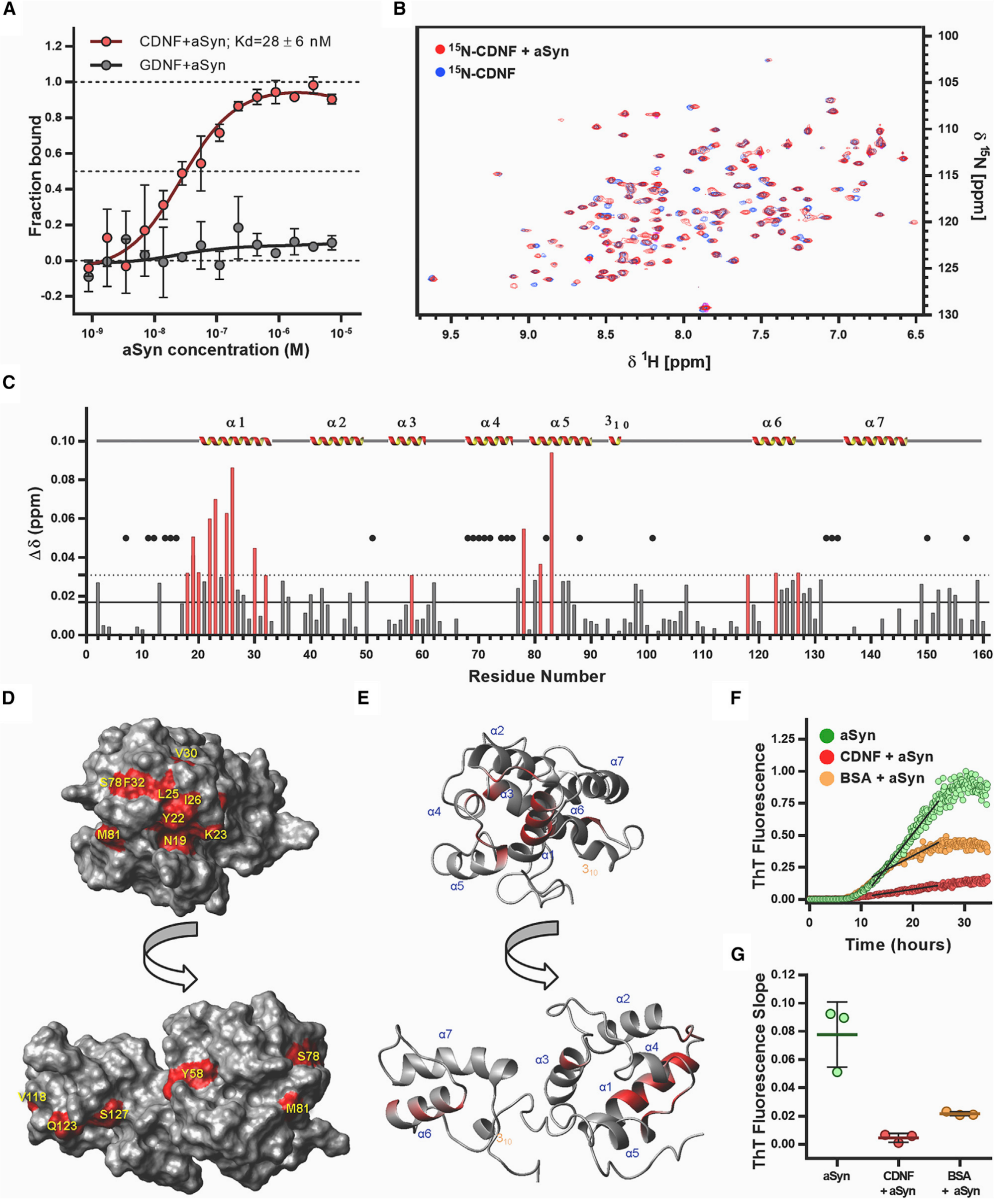
CDNF and α-synuclein (aSyn) interaction *in vitro*, sites of interaction and interaction kinetics (A) Human recombinant CDNF (n = 3) and GDNF (n = 2) proteins were used as fluorescently labeled targets at final concentrations of 20 nM in microscale thermophoresis experiments. Recombinant purified α-synuclein was used as a ligand in an indicated concentration range. Symbols and error bars represent mean values ± standard deviation. The experimental data were normalized against CDNF data, which fit to a saturation one-site binding equation at *R*^*2*^ = 0.92. K_D_ value ± standard error estimates calculated from the fit are shown in the figure legend. (B) 2D [^1^H-^15^N]-HSQC spectra of the^15^N-labeled human recombinant CDNF in the absence (blue) and in the presence (red) of monomeric α-synuclein. Spectra were collected at 25°C with 0.2 mM of each protein. (C) Bar plot of the combined chemical shift difference (Δδ) of ^1^H and ^15^N for each amino acid of CDNF after addition of monomeric α-synuclein. The mean (black line) and standard deviation (dashed line) of all Δδ (ppm) are shown. Chemical shift differences above the standard deviation are identified by red bars. The black dots represent peaks that were not found in the 2D [^1^H-^15^N]-HSQC spectra. Secondary structure elements are identified in the graph. (D) Surface plot of CDNF 3D structure highlighting residues with combined chemical shift differences (Δδ) above 0.03 ppm after addition of α-synuclein. This indicates influence of α-synuclein on these residues. (E) Cartoon of the CDNF polypeptide backbone fold at the same orientations of the surface plots, highlighting in red the locations of the residues identified in (D). (F) ThT fluorescence experiment: 70 μM of α-synuclein in the absence (green) and in the presence of 70 μM human recombinant CDNF (red) or 70 μM BSA (yellow) were incubated at 37°C under agitation in a 96-well plate with 1/8-in diameter polyballs, n = 3. All wells have 10 μM ThT. A linear regression was calculated for each curve, to represent the slope of the growth phase of ThT fluorescence (black lines). (G) The ThT fluorescence slopes of the growth phase for three experiments are plotted. Floating bars represent the limits and the mean for each group. One-way ANOVA was applied for assessing the differences between the various groups (p = 0.0013).

**Figure 3:**
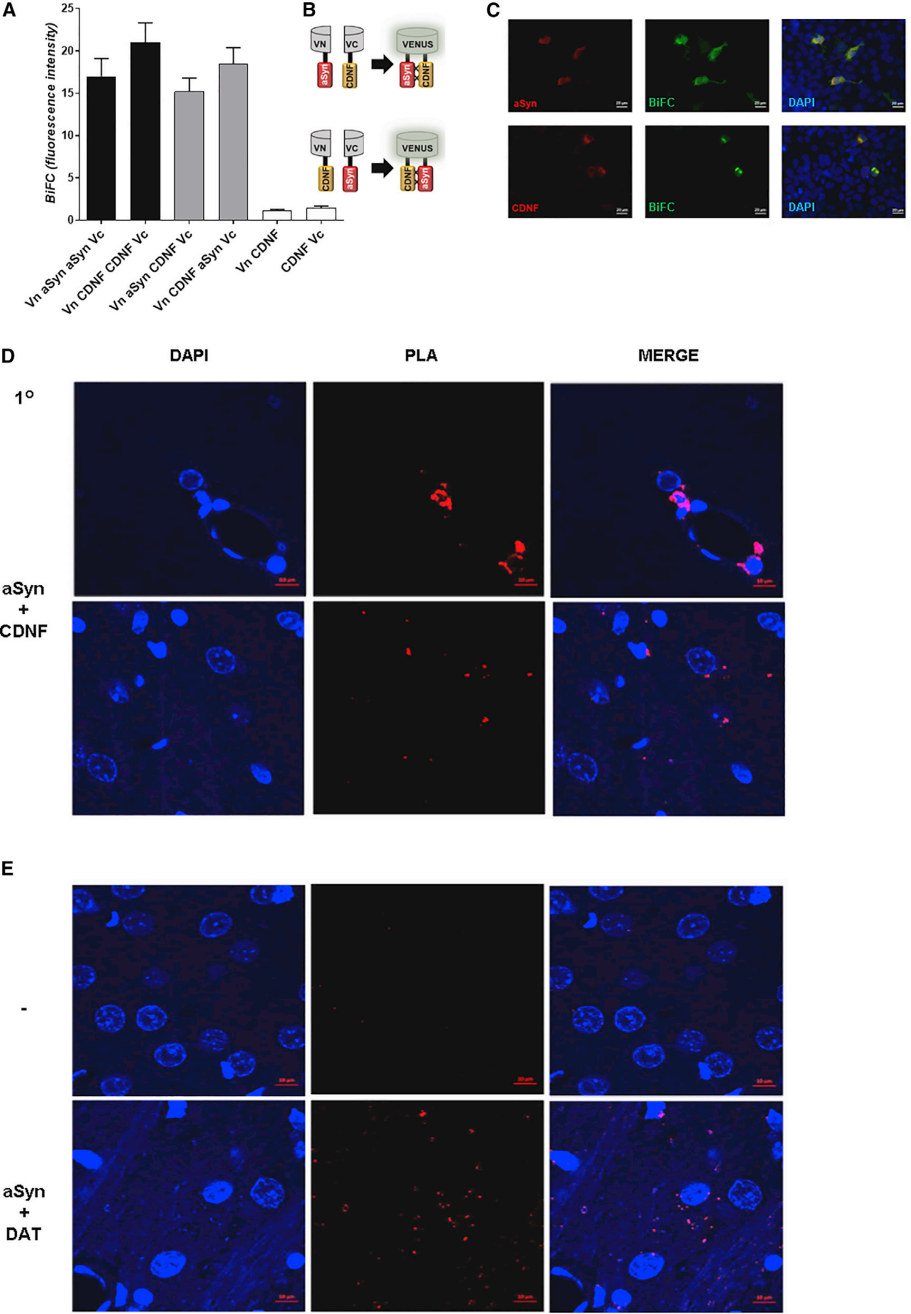
CDNF interacts with α-synuclein (aSyn) in cells and in rat brain tissue (A–C) The interaction between CDNF and α-synuclein was probed in living cells using the bimolecular fluorescence complementation (BiFC) assay. Living HEK cells expressing different α-synuclein and CDNF BiFC constructs (indicated on the left) were imaged, and the Venus fluorescence confirmed the interaction in living cells. Cells were then fixed and processed for immunocytochemistry and stained with antibodies against α-synuclein and CDNF. BiFC interactions are presented in green, counterstaining (aSyn and CDNF) is shown in red, and DAPI staining is shown in blue. Images of controls used for the BiFC studies are shown in Figure S4. Scale bar represents 20 μm. (D and E) Proximity ligation assay (PLA) was performed to verify the CDNF and α-synuclein interaction in adult 3-month-old male Wistar rats’ brains using α-synuclein and CDNF primary antibodies (aSyn + CDNF). CDNF was injected to the right striatum, and animals (n = 4) were perfused 2 h later. Representative images were taken at 63× from the striatum.Scale bar represents 30 μm. (D) The PLA signal indicating an interaction is shown in red, and DAPI staining is in blue. (E) Sections were subjected to Duolink PLA with either no primary antibodies as a negative control (−), or α-synuclein and dopamine transporter (DAT) primary antibodies for a positive control (aSyn + DAT). Error bars represent SEM.

Next, we investigated whether purified recombinant CDNF and α-synuclein proteins interact *in vitro* using microscale thermophoresis (MST). Our results show that CDNF interacts with α-synuclein following a saturation one-site binding curve with a K_D_ of 28 ± 6 nM (Figure 2A). We also tested glial cell line-derived neurotrophic factor (GDNF), and our data indicated that there is no specific interaction between GDNF and α-synuclein (Figure 2A). The interaction of CDNF with α-synuclein was then characterized by nuclear magnetic resonance (NMR), monitoring the amide ^1^H,^15^N chemical shifts of ^15^N-labeled CDNF. The two-dimensional (2D) [^1^H,^15^N]-HSQC (heteronuclear single quantum coherence) spectra of ^15^N-labeled CDNF are shown overlaid in the absence (blue) and presence (red) of α-synuclein (Figure 2B). The spectra showed prominent variations in the chemical shifts of some peaks, but no alterations for others, a well-described effect related to the interaction between proteins. We found that the significant changes of chemical shifts are grouped in three regions (Figure 2C): on the helix α1 with many amino acids from L18 to F32; around helix α4 and α5 with residues S78, M81, and A83, both regions at the N-terminal domain of CDNF; and on helix α6 with residues V118, Q123, and S127, at the C-terminal domain. Some amino acid residues do not present backbone amide signals due to intermediate conformational dynamics already observed by our group elsewhere.^20^ These residues are not tightly packed into the hydrophobic core of CDNF, and, because of that, they undergo conformational exchange on the order of milliseconds. This exchange rate is close to the NMR chemical shift difference of the involved residue at the distinct CDNF conformations, which leads to extensive NMR line broadening with concomitant extreme reduction in intensity. Due to this, we cannot detect any effect on these amino acids (black dots in Figure 2C). We then selected the amino acids that underwent significant changes of chemical shifts and analyzed their accessibility to the solvent on the surface of the CDNF structure (PDB: 4BIT; Figures 2D and 2E; summarized in Table S2). Many of these amino acids are grouped in a contiguous region on CDNF, with a surface area of 509.7 Å^2^ (residues L18, N19, R20, Y22, K23, L25, I26, V30, F32, S78, and A83). Residues L18 and A83 are mostly buried in contact to each other and are not depicted on the surface plot. Residues Y58 and M81 are the only ones that are on the other side of the CDNF structure, shown in Figures 2D and 3E, and might represent an overall conformational switch in CDNF upon α-synuclein binding. To evaluate the interactions from the perspective of α-synuclein, we carried out the 2D [^1^H,^15^N]-HSQC experiments of ^15^N-labeled α-synuclein, and we did not identify significant differences in the NMR spectra of ^15^N-labeled α-synuclein upon the addition of CDNF (Figure S1).

### CDNF inhibits the aggregation of recombinant α-synuclein *in vitro*

To investigate whether CDNF could directly interfere with the aggregation of α-synuclein, we performed *in vitro* aggregation kinetic assays using purified proteins. Recombinant α-synuclein was incubated with or without CDNF or bovine serum albumin (BSA) at 37°C in a 96-well plate with 1/8-in diameter polyballs under shaking. The aggregation kinetics were monitored using Thioflavin T (ThT) (Figure 2F). We found an inhibition of the α-synuclein aggregation by CDNF (red dots). The aggregation was also inhibited by BSA as previously described,^26^ but not as efficiently as in the presence of CDNF. CDNF inhibited the growth phase of the α-synuclein aggregation, as shown by a substantial change in the slope of the growth phase (Figures 2F and 2G). Neither CDNF nor BSA alone interfered with ThT signal (data not shown).

We performed cell viability assays on human H4 neuroglioma cells to determine the effect of 1‒10 μM CDNF on the toxicity induced by α-synuclein oligomers or fibrils. After treatment with CDNF for 1 h, 10 μM α-synuclein oligomers or fibrils were applied (considering the monomeric form for the concentration assay). Pretreatment with CDNF protected cells against the toxicity of α-synuclein oligomers in a dose-dependent manner, but not against fibrils (Figure S2), suggesting that CDNF activity is specific to earlier species of α-synuclein oligomers. However, the samples of oligomers and fibrils used contain a mixture of different species (Figure S3).

### α-synuclein and CDNF interact in living cells and rat brains

To assess how CDNF interacts with α-synuclein in a cellular milieu, we performed the bimolecular fluorescence complementation (BiFC) assay in living cells (Figures 3A–3C). First, we transfected human embryonic kidney (HEK) cells and analyzed the reconstitution of the Venus fluorophore 48 h after transfection, using epifluorescence microscopy. Since we detected green fluorescence signal, confirming the interaction, we fixed the cells and performed immunocytochemistry using anti-CDNF and anti-α-synuclein antibodies to confirm the expression of the two proteins (Figure 3C). We also co-expressed VN-α-synuclein (N-terminal part of Venus-α-synuclein) with α-synuclein-VC (C-terminal part of Venus-α-synuclein), and VN-CDNF with CDNF-VC, and found robust BiFC signal from these homomeric combinations (Figures 3A and S2). As negative controls, we cotransfected cells with VN-CDNF and VC, or with VN with CDNF-VC. These combinations yielded minimal background signal (Figures 3A and S2).

To analyze interaction *in vivo*, we used the *in situ* proximity ligation assay (PLA) to discover whether interaction of α-synuclein and CDNF proteins was occurring in the rodent brain. Adult 3-month-old rats were injected with CDNF to the striatum, perfused 2 h later, and the brains were analyzed using an antibody specific to CDNF and an antibody that recognizes endogenous α-synuclein. For the positive control, an antibody to dopamine transporter (DAT) was used with the α-synuclein antibody, since a DAT-α-synuclein interaction has been previously observed in a similar assay.^27^ For the negative control, no primary antibodies were used. We detected fluorescence signal at 568 nm, indicating an interaction according to the PLA for α-synuclein and injected CDNF (Figure 3D) as well as for the endogenous DAT and α-synuclein proteins (Figure 3E).

### CDNF reduces internalization of preformed α-synuclein fibrils to primary neurons

**Figure 4:**
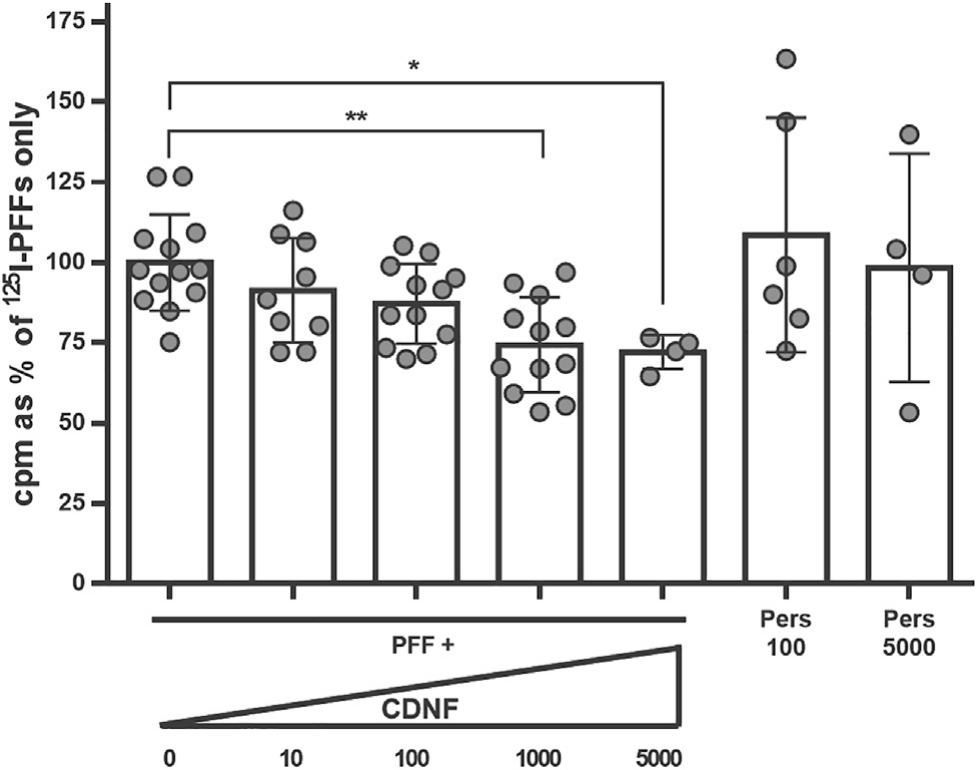
CDNF reduces internalization of α-synuclein preformed fibrils (PFFs) in a dose-dependent manner ^125^I-labeled α-synuclein PFFs were added to mouse hippocampal cultures (E16, DIV 7) together with CDNF at different concentrations (ng/mL) or persephin (ng/mL) as a negative control. The PFFs were allowed to be internalized into neurons for 60 min at 37°C, after which the cultures were washed twice with PBS and finally with acid wash that removes the unspecifically bound extracellular PFFs. After this, the cells were lysed with 1 N NaOH, and the radioactive signal from the internalized PFFs was counted using gamma counter. Kruskal-Wallis test = 18.79, number of groups = 7, p = 0.0045. Error bars represent SEM.

Next, we wanted to test whether human CDNF can affect the internalization of fibrillary α-synuclein to neurons. For this, we added ^125^I-labeled α-synuclein PFFs to hippocampal cultures together with different concentrations of CDNF. CDNF was able to dose-dependently reduce the number of fibrils internalized by neurons in 60 min, although not by more than 25% (Figure 4; p < 0.01).

### CDNF enhanced the formation of pSer129-α-synuclein inclusions but decreased the total amount of insoluble pSer129-α-synuclein in neuron primary culture

**Figure 5:**
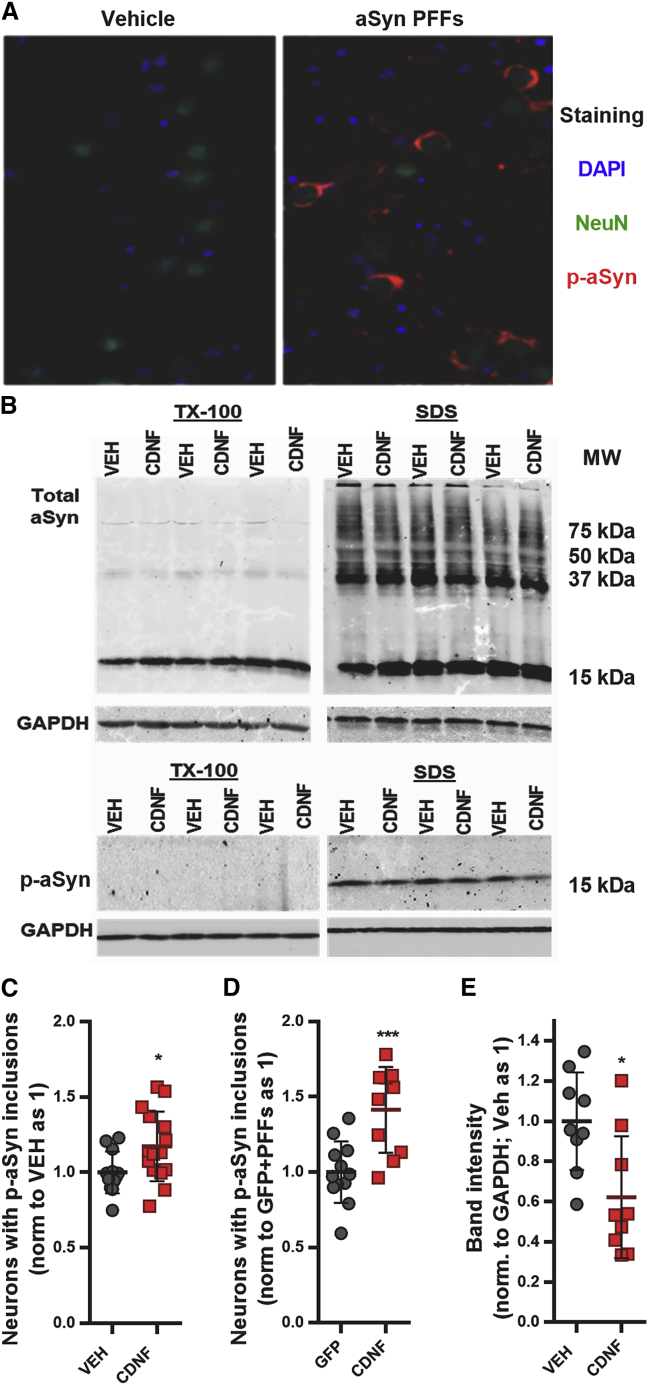
CDNF increases the number of neurons carrying pSer129-α-synuclein inclusions and decreases the amount of insoluble pSer129-α-synuclein in primary neuronal culture Timelines of experiments are shown in Figure S5. (A) Representative scanned image of hippocampal culture in 96-well plate seeded with α-synuclein PFFs at days *in vitro* (DIV 7) and fixed 7 days post-seeding (DIV 14) for immunostaining with anti-NeuN and anti-pSer129 α-synuclein antibodies. (B) Cortical cultures were seeded with PFFs at DIV 7 and treated at DIV 10 with CDNF (125 ng/mL) or PBS as vehicle (VEH), and at DIV 12, the proteins were isolated sequentially with Triton X-100 buffer followed by SDS buffer, separated by PAGE-SDS, and visualized by immunoblotting. (C) The effect of CDNF (1 μg/mL) pretreatment on the number of neurons carrying pSer129-α-synuclein inclusions after PFF seeding (unpaired t test, t = 2.276 df = 25, p = 0.032). (D) The effect of lentiviral-vector-mediated overexpression of CDNF (GFP overexpression as control) on the PFF-seeded neuronal inclusion pathology (unpaired t test, t = 4.239 df = 21, p = 0.0004). (E) The levels of p-aSyn in VEH and CDNF treated neurons quantified from the SDS immunoblots (unpaired t test, t = 2.920 df = 16, p = 0.01). Error bars represent SEM.

We used the α-synuclein PFF seeding model on primary neuronal cultures to induce the α-synuclein aggregation and analyze the progressive Lewy body-like pathology and the effect of CDNF on it (Figure S5). Seven days after PFF seeding, some of the hippocampal neurons had formed inclusion-like structures in their soma detected by antibodies against pSer129-α-synuclein (Figure 5A). CDNF, when added 1 h before PFFs into the culture media, led to a significant increase in the number of neurons carrying inclusions, when compared to phosphate-buffered saline (PBS) (Figure 5C; p < 0.05). The neuronal survival was not compromised by the PFF-seeded α-synuclein aggregation, as the counts of NeuN-positive cells in the cultures were not changed (Figure S6A).

When we used lentivirus to overexpress GFP or CDNF in primary hippocampal cultures, we observed similar results. At day seven post-PFF seeding, CDNF treatment led to an increase in the number of neurons with pSer129 α-synuclein inclusions, in comparison to GFP-treated (Figure 5D; p < 0.001). Similarly, there was no change in the total number of neurons (Figure S6B).

We also studied whether CDNF could affect the total level of pSer129-α-synuclein in PFF-seeded neurons. We followed the protocol described^5^ and isolated sequentially the more (TX-100 fraction) and the less (SDS fraction) soluble proteins from primary cortical cultures after seeding them with PFFs. An antibody against total α-synuclein detected a monomer-sized band in the TX-100 fractions and in the SDS fractions, and it also detected a “smear” with lower electrophoretic mobility (Figure 5B), but these forms of α-synuclein were not affected by CDNF treatment (Figure S7). However, the amount of pSer129-α-synuclein that was detectable only in the SDS fraction at a size corresponding to the monomer was reduced by CDNF treatment (Figures 5B and 5E; p = 0.01).

### Exogenous α-synuclein fibrils seed endogenous α-synuclein to form pSer129 inclusions in rodent brains

**Figure 6:**
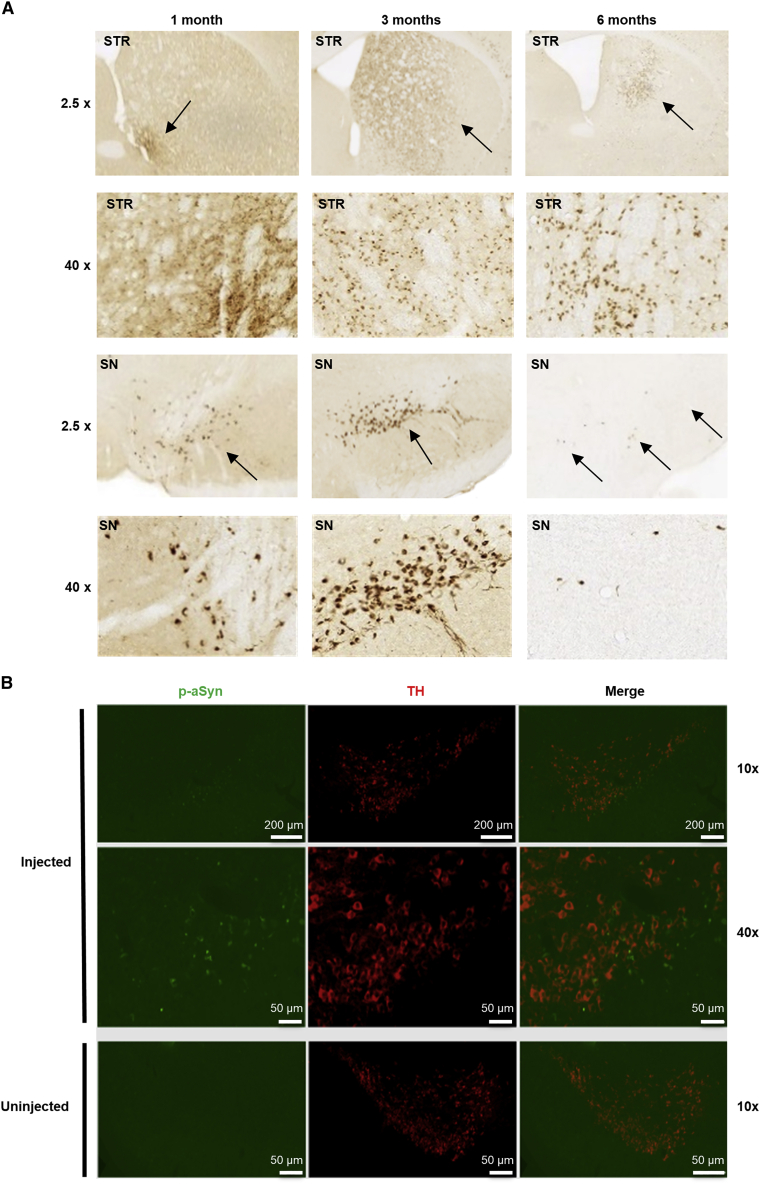
PFF-induced α-synuclein phosphorylation and propagation of α-synuclein aggregation (A) Wild-type mice (3 months old at time of injection) were injected with PFFs to the striatum (STR) and stained for α-synuclein phosphorylated at S129 (p-aSyn) in the STR and *substantia nigra* (SN) areas at 1, 3, and 6 months after PFF injection. Images at 2.5× magnification and close-ups at 40× magnification are shown. Black arrows at 2.5× indicate approximate areas of p-aSyn-positive staining (dark brown); 40× images show widespread p-aSyn-positive staining. (B) Double immunofluorescence staining of *substantia nigra* area for tyrosine hydroxylase (TH, red) and p-aSyn (green) of injected (left panels) and uninjected hemispheres (right panels). Scale bars represent 200 μm (top row) and 50 μm (bottom rows). Additional controls are included in Figure S9.

Three- or 12-month-old mice and 1-year old rats were used for the *in vivo* α-synuclein PFF experiments. The animals were intrastriatally injected with mouse α-synuclein PFFs (mPFFs), and 1 month later CDNF was given intrastriatally either as a single injection to the mice or chronically infused to rats using minipumps (timeline of experiments shown in Figure S8). Motor performance was followed throughout the experiments until the rodents were euthanized and brains collected for analysis. As reported previously,^6^^,^^28^, ^29^, ^30^ we observed spreading of phosphorylated pSer129-positive inclusions from the injection site to anatomically connected areas (Figure 6A). One month after mPFF injection, there was pSer129 immunoreactivity around the injection site in the striatum. Three months after mPFF injection, the pSer129 immunoreactivity in the striatum was covering a larger area. Six months after mPFF injection, there were fewer inclusions in the striatum, and overall immunoreactivity was more spread around the brain areas. Importantly, there was phosphorylated α-synuclein present in the *substantia nigra pars compacta* at all time points measured. This indicates that α-synuclein was successfully seeded and spread from the striatum to the *substantia nigra* after injection of the mPFFs. We did not observe pSer129-positive immunoreactivity in either the PBS-injected group at 6 months post-injection or in the α-synuclein knockout animals at 1 month post-injection (Figure S9). Immunoreactivity to pSer129-α-synuclein localized in tyrosine hydroxylase (TH)-positive cell bodies and neurites in the *substantia nigra pars compacta* and reticulata, respectively (Figure 6B).

### Injection of α-synuclein PFFs to mice results in behavioral deficits that can be ameliorated by CDNF

**Figure 7:**
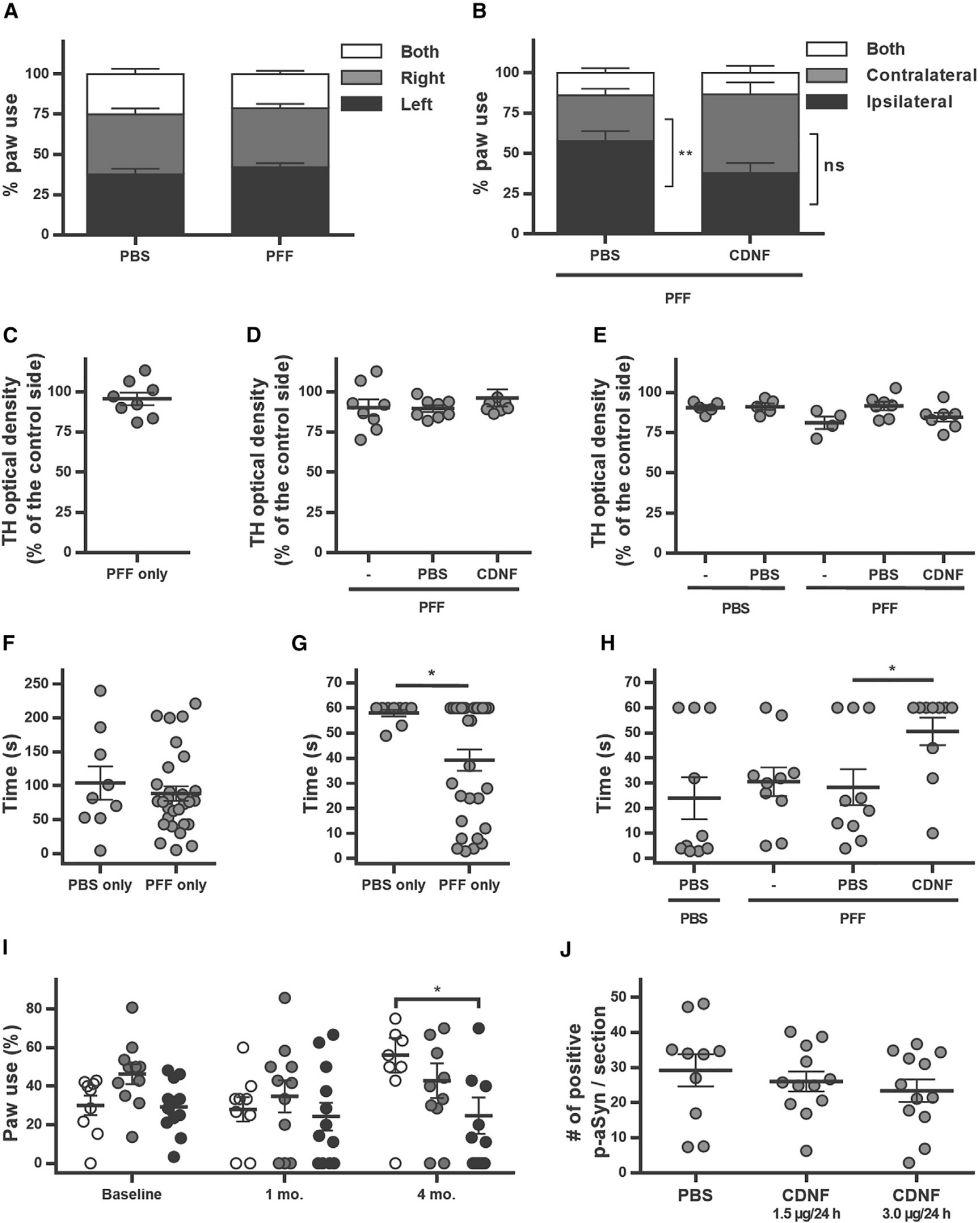
Behavior and outcome after CDNF treatment in PFF-injected mice and rats (A–E) Mouse behavior and TH optical density after injections to 3-month-old mice. (A) Cylinder test at 1 month after PFF injection, before CDNF treatment, n = 8/group. (B) Cylinder test at 3 months after PFF/PBS injection, 2 months after CDNF/PBS. The graph represents ipsilateral, contralateral, as well as both paw touches on the cylinder wall. PFF + PBS/PFF + CDNF indicates that PFFs were given at day 0 and PBS/CDNF was given 1 month later, n = 8/group. p = 0.0051 (C) TH optical density in the striatum as a percentage of the control side at 1 month after PFF injection, n = 8. (D) TH optical density in the striatum as a percentage of the control side 3 months after PFFs. PFF only indicates PFFs given at day 0, and PFF + PBS/CDNF PFFs given at day 0 and PBS/CDNF 1 month later, n = 8/group. (E) TH optical density in the striatum as a percentage of the control side. PBS/PFF only indicates PBS or PFFs given at day 0 and no other injections, PBS + PBS indicates PBS given at day 0 and again 1 month later, PFF + PBS/CDNF indicates fibrils at day 0 and PBS/CDNF 1 month later, n = 4–7/group. (F–H) Mouse behavior after injections to 1-year-old mice. (F) Rotarod 1 month after PBS (control, n = 10) or PFF injections (n = 30). (G) Coat hanger time 1 month after injection, PBS (n = 9); PFF (n = 30). p = 0.022. (H) Coat hanger time 3 months after PBS or PFFs injection, 2 months after PBS or CDNF. PBS/PFF only indicates PBS or PFFs given at day 0 and no other injections, PBS + PBS indicates PBS given at day 0 and again 1 month later, PFF + PBS/CDNF indicates fibrils at day 0 and PBS/CDNF 1 month later, n = 10/group. p = 0.0410. (I and J) Behavior and histopathological outcome after injections to 1-year-old rats. (I) Contralateral paw use (percent of total paw use). Baseline indicates the data obtained before PFFs were injected, 1 month is 1 month after fibrils but before installation of minipumps, and 4 months is 4 months after fibrils and 3 months after treatment. Minipumps administer PBS (white symbols), CDNF at 1.5 μg/day (gray symbols), or 3.0 μg/day (black symbols) to the striatum for 30 days, n = 9–12/group. p = 0.0114. (J) Number of inclusions of α-synuclein phosphorylated at S129 (p-aSyn) in the *substantia nigra* for each treatment 4 months after fibril injections, n = 10–12/group. Error bars represent SEM.

Mice at 3 months or 1 year of age were injected with mPFFs or PBS, and behavioral assessment was performed at 1, 3, and 6 months post-mPFF injection (experimental timeline shown in Figure S8). At 1 month after mPFF injection, PBS or CDNF was injected. At 1 month post-mPFFs, the younger mice did not show any significant difference compared to PBS in the cylinder test on paw use (Figure 7A; two-way ANOVA, F [2, 174] = 0.9329, p = 0.3954) or in the level of TH optical density (Figure 7C; average = 95.55%, SEM = 3.92%). At 3 months after mPFF injections, 2 months after CDNF or PBS, younger mice injected with PBS used the ipsilateral paw significantly more than the contralateral, whereas CDNF-treated animals used both paws more evenly (Figure 7B; two-way ANOVA, column factor F [2, 42] = 21.51, p < 0.0001. Tukey’s multiple comparison test as post hoc, PBS ipsilateral versus PBS contralateral, p = 0.0051; CDNF ipsilateral versus CDNF contralateral, p = 0.7109). No significant change on TH optical density was observed among these three groups (Figure 7D; one-way ANOVA, F [2, 21] = 0.6828, p = 0.5161), as previously shown.^6^ At 6 months there were also no significant differences between the groups on TH-positive fiber density (Figure 7E; one-way ANOVA, F [4, 23] = 2.782, p = 0.0508). For older mice, at 1 month post-PFFs there was no significant difference on rotarod between PBS- and mPFF-injected mice (Figure 7F; unpaired t test, t = 0.7135 df = 38, p = 0.4799). However, in the coat hanger test, we detected significant differences between PBS and mPFF groups (Figure 7G; unpaired t test, t = 2.390 df = 37, p = 0.022). At 3 months after PFFs, 2 months after PBS/CDNF injections, older mice injected with CDNF performed significantly better than the ones treated with PBS (Figure 7H; one-way ANOVA, F [3, 36] = 3.046, p = 0.0410. Tukey’s multiple comparison post hoc test: PFF + PBS versus PFF + CDNF, p = 0.0405).

### Chronic CDNF infusion prevents PFF-induced changes in paw use

Due to promising results with one intrastriatal CDNF injection in mice, we set out to study whether a chronic infusion of CDNF would be even more efficient therapeutically in 1-year-old rats seeded with mPFFs. mPFFs were injected into the striatum, and 1 month later CDNF was given via minipump to the striatum at 1.5 μg/day or 3 μg/day for 30 days, with PBS as vehicle control. The cylinder test was used as an outcome behavioral measure. At baseline (before fibril injection) and 1 month (before minipump installation) there were no significant differences between treatments over time for contralateral paw use (Figure 7I; two-way ANOVA, treatment: F [2, 58] = 2.788, p = 0.0698; time: F [2, 29] = 2.448, p = 0.1041; interaction: F [4, 58] = 2.048, p = 0.0995). At 4 months, however (2 months after minipump installation), there was a significant difference between PBS and CDNF 3 μg/day on contralateral paw use (two-way ANOVA, Tukey’s post hoc test for multiple comparisons, p = 0.0114). Four or 5 months after mPFF injection, brains were removed and analyzed for the number of pSer129-α-synuclein inclusions in the *substantia nigra*. There were no significant differences between treatments (Figure 7J; one-way ANOVA, F [2, 31] = 0.6597, p = 0.5241).

## Discussion

CDNF has a C-terminal KTEL retrieval sequence, indicating that CDNF is mostly, but not exclusively, an ER-luminal protein. This indicates that CDNF is not a conventional neurotrophic factor, acting intracellularly and when secreted. The presence of CDNF in the ER is associated with a protective effect against ER stress inducers and concomitant modulation in the expression levels of unfolded protein response (UPR) signaling proteins.^31^^,^^32^ Moreover, MANF, the paralog of CDNF, functions as a chaperon in the ER.^33^^,^^34^ Both α-synuclein monomers and oligomers can accumulate in isolated fractions of ER in animal PD models and in brains from PD patients.^35^^,^^36^ Also, α-synuclein interacts with ER-related proteins mediating further toxic events, inducing the UPR and culminating with neuron death in PD.^37^^,^^38^ This indicates the possibility of α-synuclein and CDNF association in this organelle.

Considering this context of intracellular mechanism of action, it is puzzling that exogenously added CDNF protects cells from toxic molecules including α-synuclein. The amount of CDNF secreted from neurons compared to MANF is low,^39^ suggesting limited chances for endogenous CDNF to interact with extracellular or cytoplasmic α-synuclein. However, we have shown previously that intrastriatally infused exogenous CDNF is endocytosed, and this can be one of the mechanisms that lead to its neuroprotective effect.^40^ In parallel, extracellular CDNF causes a significant reduction in the cellular uptake of α-synuclein PFFs, which is compatible with an extracellular mechanism of action of CDNF.

Interestingly, we found a direct inhibitory effect of CDNF on α-synuclein aggregation, mediated by its high-affinity association with α-synuclein monomers. Although we identified an interaction patch of α-synuclein on the surface of CDNF, our NMR experimental setup was unable to detect any interaction patch on α-synuclein. This is most likely because under the conditions tested, we see only 90 HN peaks out of 152 expected for human α-synuclein. This indicates that the interaction site in α-synuclein includes amino acids that have strong HN exchange with buffer, have intermediate conformational dynamics and/or residues with overlapped HN (amide proton (HN)) signal, common features in this intrinsically disordered protein.^41^ Another possible explanation is that CDNF interacts with an oligomer of α-synuclein, which we cannot say the precise size of but is large enough to have low molecular tumbling rate in solution. Using the current experimental setup, such protein oligomers do not present NMR signals due to strong spin-spin relaxation and consequent extreme linewidth broadening. A similar result has been described elsewhere.^42^

During the *in vitro* aggregation study, CDNF inhibits the growth phase of α-synuclein aggregation, where it is considered to present a higher concentration of misfolded oligomeric species. Since misfolded oligomers are considered a toxic form of α-synuclein both *in vitro*^43^ and *in vivo*,^44^ our data suggest that CDNF might protect cells, at least partially, by interfering with the formation of toxic oligomeric α-synuclein intermediates. This also aligns with our results showing that PFF uptake is reduced by approximately 25% with CDNF application, where CDNF is having a stronger effect on oligomeric species as opposed to the fibrillar form. Moreover, our data suggest that the inhibitory effect of CDNF on α-synuclein aggregation can be achieved when CDNF is localized in the ER lumen where intracellular inhibition of α-synuclein auto-association is most likely caused by UPR modulation. However, the inhibition of α-synuclein intracellular aggregation by purified CDNF added to the culture medium may account for a distinct molecular mechanism. Extracellular CDNF might act to some extent by binding to α-synuclein or by decreasing the intake of α-synuclein via plasma membrane receptors responsible for α-synuclein uptake, such as heparan sulfate^45^ or LAG3.^46^ Also, CDNF infusion into the striatum has been shown to modulate UPR signaling in stressed cells,^47^ and CDNF added to culture media of stressed neurons has been shown to modulate ER stress,^48^ which suggests an indirect effect of CDNF on α-synuclein aggregation intermediated by ER stress modulation. Therefore, the reason we observe inhibition of α-synuclein oligomerization and a modest effect on fibril uptake by CDNF, but increased number of pSer129 inclusions in the soma of neurons and no discernible effect on these inclusions at the time point measured in rodent brains, likely has to do with the localization of endogenous CDNF or timing of exogenous CDNF administration. The details of these mechanisms warrant more study.

The pSer129-α-synuclein is the most abundant component of Lewy bodies,^49^ and the fact that CDNF can reduce the overall levels of insoluble pSer129-α-synuclein in neuronal cultures is in line with its proposed neuroprotective activity. Although the exact mechanisms and consequences of α-synuclein phosphorylation are unknown, it is thought to be linked with pathological aggregation. In the postmortem PD brain, aggregated α-synuclein has been found both in neurites and cell bodies.^4^ While somatic Lewy bodies have been suggested to be an adaptive measure in neurons,^50^ in later stages they likely reflect a defect of aggregated protein clearance that becomes detrimental when the cell soma accumulates enough Lewy bodies.^51^ Interestingly, we found more neurons carrying large soma inclusions after CDNF treatment or overexpression, while the neuron survival was not affected by the PFFs or CDNF treatments.

*In vivo*, protection from the PFF seeding-induced functional deficit was observed when a single injection of recombinant human CDNF was given to mice or continuous infusion of CDNF to aged rats, 1 month after PFFs. Even though we detected pSer129-α-synuclein inclusions in the rodent brains after PFF seeding, we did not detect effects of CDNF on the number of these inclusions in the *substantia nigra*. Therefore, it seems possible that even with a direct interaction with α-synuclein, it is rather more likely, as evidenced from our studies *in vitro*, that CDNF is affecting the localization and the kinetics of association of α-synuclein to the aggregates and not its total levels. This outcome could be more important, since α-synuclein is a protein present in healthy human brains and therefore a therapy should not deplete its levels. This may be dependent on the time point of both CDNF administration and experiment endpoint, since CDNF was administered 1 month after PFF injections and the experiments lasted several months. Therefore, based on our current *in vivo* results where we did not observe any differences between the number of pSer129-α-synuclein inclusions, we cannot conclude that the mechanism of action of CDNF is directly related to inclusion formation without including more time points.

If CDNF is inhibiting the formation of the toxic species of α-synuclein during the growth phase, we must ask why we still observe Lewy body-like structures in neurons and in the mouse brain. There is evidence of Lewy body-like inclusions being the endpoint event, and neurons bearing inclusions eventually die.^52^ From the current state of the literature, it seems that intermediate oligomers form fibrils and that α-synuclein fibrils form Lewy body-like inclusions *in vitro* and *in vivo*.^53^ Interestingly, 3-[4,5-dimethyltriazol-2-yl]-2,5 diphenyltetrazolium bromide (MTT) assays performed with pre-treatment of the cells with CDNF followed by addition of α-synuclein enriched in oligomers or fibrils shows a protective effect of CDNF against the oligomeric fraction but not against the fibrillar fraction of α-synuclein. The toxicity of α-synuclein oligomers and fibrils is still a matter of debate and is likely to involve different cellular pathways.^54^^,^^55^ Since we did not observe loss of TH here or neuron loss *in vitro*, we cannot conclude that the inclusions were causing cell death, but perhaps only a functional deficit, as evidenced from the behavioral data. Studies with longer time points might be needed to observe significant cell loss, as with a Parkinson’s patient.

Assuming that oligomers are the most toxic form of α-synuclein leading to fibrillar Lewy body formation, our data suggest that CDNF protects cells, at least partially, by interfering with the formation and/or clearance of those toxic oligomeric intermediates. Based on that, we hypothesized that CDNF prevents the early toxic aggregate population (oligomers) but does not have a significant effect on fibril preparations. Therefore, timing of application of PFFs and CDNF administration may differentially affect the inclusion formation. For example, earlier administration of CDNF may reduce the formation of the toxic aggregates, resulting in fewer pSer129-α-synuclein inclusions *in vivo* and a more robust behavioral outcome, but this would need further study. However, what is also needed is the identification and separation of oligomeric and fibril species and studies on which α-synuclein misfolded forms are the most detrimental ones. Nevertheless, our findings help to describe the effects of exogenous CDNF in therapeutic contexts, and the effects of CDNF demonstrated here stimulate further investigation on the mechanism of action of this unusual neurotrophic factor.

## Materials and methods

A list of primary antibodies used in this work is available in Table S1.

### In vitro

#### PCA

PCA based on split humanized GLuc^22^ was performed as previously described for α-synuclein oligomerization.^23^ Neuro2A cells were plated at a density of 10,000 cells per well on poly-L-lysine-coated 96-well plates (PerkinElmer Life Sciences, white wall-clear bottom). Twenty-four hours post-plating, 125 ng of total plasmid DNA was transfected per each well using JetPei transfection reagent (Polyplus) according to the manufacturer’s instructions. The amount of CDNF expression plasmid per well was 10% (12.5 ng), 30% (37.5 ng), and 50% (62.5 ng) of total plasmid DNA. Mammalian expression vectors (pCR3.1 and pcDNA3; both with CMV promoter) were used to coexpress CDNF with its original N-terminal signal sequence, sRAGE, and 14-3-3ζ with the PCA reporter plasmids. In addition, pRC/CMV/βGal and pCMV-GLuc plasmids were used to express β-galactosidase and full-length GLuc, respectively. All expression vectors were encoding for human proteins, except for β-galactosidase and GLuc. PCA signal was measured 48 h post-plating. Cell culture medium was changed to phenol red- and serum-free Dulbecco’s modified Eagle’s medium (DMEM) (Invitrogen) for 30 min before the measurement. Native coelenterazine (Nanolight Technology) was injected to a final concentration of 20 μM per well, and bioluminescence PCA signal was detected by Varioskan Flash multiplate reader (Thermo Scientific). For each experimental condition, three or more independent experiments were performed at a replicate of four wells per experimental condition. Chemicals and drugs were used at indicated concentrations. Dimethyl sulfoxide was used as a vehicle control.

#### Effect of CDNF on α-synuclein aggregation in human H4 cells

H4 human neuroglioma cells (ATCC HTB-148) were cultured in monolayer in 75 cm^2^ bottles and incubated under 5% CO_2_ atmosphere at 37°C. The culture medium used was Gibco Opti-MEM supplemented with 10% (v/v) fetal bovine serum (FBS) (Cultilab) and 1% penicillin/streptomycin antibiotics. For cell transfection, H4 cells were seeded in 6-well plates at a density of 75,000 cells/well for 16 h containing Opti-MEM culture medium supplemented with 10% (v/v) fetal bovine serum (Cultilab) and 1% penicillin/streptomycin antibiotics. On the day of transfection, Metafectene transfection reagent (Thermo Fisher) was added in serum-free Opti-MEM medium and incubated for 5 min. The plasmid of interest and Metafectene reagent were mixed at a ratio of 1:3. The maximum amount of DNA added was 4 μg, and reagents were incubated for 30 min. Finally, the mixture was added to each well and incubated for 48 h in Opti-MEM culture medium supplemented with 10% (v/v) fetal bovine serum (Cultilab) and 1% penicillin/streptomycin antibiotics. Cells were transfected with SynT/Synphilin-1 and CDNF with its original N-terminal signal sequence, or with SynT/Synphilin-1 and then treated with recombinant CDNF produced in bacteria as described elsewhere.^20^ Immunocytochemistry assays were performed to study α-synuclein inclusions. Cells were washed twice with PBS and fixed with 4% paraformaldehyde (PFA) for 10 min at room temperature (RT). After three washes with PBS, cells were permeabilized with 0.5% Triton X-100 (Sigma-Aldrich) for 20 min. After blocking in 5% BSA diluted in Tris-buffered saline (TBS) for 1 h, each well was incubated with primary antibody (anti α-synuclein mouse, 1:5,000, BD Transduction Laboratory and/or rabbit anti-CDNF 1:2,000, Sigma) overnight followed by secondary antibody (IgGAlexa488 anti-mouse and/or IgG-Alex 568 anti-rabbit, 1:10,000, Invitrogen) for 2 h. Finally, cells were stained with DAPI (1:5,000 in PBS, Molecular Probes) for 5 min, washed, and kept in PBS for fluorescence microscopy (Olympus IX81-ZDC microscope system), with a 20× objective. A total of three independent experiments were performed and around 25 images were randomly taken out of each condition for quantification of α-synuclein inclusions. The percentage of cells presenting α-synuclein inclusions was then determined by counting at least 100 cells for each treatment using ImageJ software and scored based on the α-synuclein inclusion pattern and classified into four groups: cells without inclusions, less than five inclusions (1–4 inclusions), between five and nine inclusions (5–9 inclusions), and more than 10 inclusions (>10 inclusions). Cells classified in the last group (more than 10 inclusions) can sometimes present numerous α-synuclein inclusions in the cytoplasm, making it impossible to count the maximum number observed. Results were expressed as a percentage of the total number of transfected cells. All the images were taken in a blinded manner, where the samples were processed without knowledge of the conditions tested. For that, the samples submitted to microscopy were randomized and labeled with numbers.

#### MTT cytotoxicity assay

For the formation of aggregates, recombinant human α-synuclein was incubated at 140 μM in PBS (pH 7.4) at 37°C under constant and moderate shaking for 48 h or 7 days until oligomers and amyloid fibers formed, respectively, as previously described.^20^ The transition of α-synuclein from initial soluble monomeric form to aggregated state was determined by measuring light scattering in a Jasco FP-8200 spectrofluorometer (Jasco, Easton, MD, USA) with an excitation wavelength of 330 nm and emission range from 320 to 340 nm at 25°C. Final protein concentration was 10 μM in native buffer. Solutions without protein were used as negative controls. For the cytotoxicity assays, the H4 cells were seeded in 96-well plates at a density of 25,000 cells/well for 16 h in Opti-MEM culture medium supplemented with 10% (v/v) fetal bovine serum (Cultilab) and 1% penicillin antibiotics. Subsequently, the cells were pretreated with vehicle and different concentrations of CDNF for 1 h and then challenged with 10 μM of oligomeric or fibrillar α-synuclein. After 24 h, 20 μL MTT (5 mg/mL in PBS) was added to the cells, including controls, and incubated for 3 h at 37°C. The medium was carefully removed to preserve cells, and 100 μL of DMSO was added to dissolve the forming crystals. Absorbance was measured at 570 nm, and a wavelength of 630 nm was used as a reference for discounting any turbidity interference.

#### MST assay

The binding affinities of recombinant proteins were measured by MST using Monolith NT.115 Instrument (NanoTemper Technologies). All measurements were performed at 25°C, at medium MST power. The LED power was set to 50%. Recombinant human CDNF protein (produced in mammalian protein expression system, Biovian, Turku, Finland)^40^ and recombinant human GDNF (P-103-100, Icosagen, Tartu, Estonia) were both expressed and purified from Chinese hamster ovary (CHO) cells and labeled using the amine-reactive Monolith Protein Labeling Kit RED-NHS 2^nd^ Generation kit (L011, NanoTemper Technologies). Removal of free, unreacted dye from proteins after the labeling reaction was done using Zeba Spin Desalting Columns (89882, Thermo Fisher Scientific) according to the manufacturer’s instructions. Final concentrations of labeled proteins in interaction measurements were kept constant at 20 nM. Recombinant human α-synuclein (S-1001-1, rPeptide, Watkinsville, GA, USA) produced in *Escherichia coli* was used as a ligand at indicated concentrations. All experiments were done using Monolith NTT premium coated capillaries (K005, NanoTemper Technologies), 14 capillaries in each measurement. Three independent measurements were done for each binding pair. All experiments were done in MST buffer (10 mM Na-phosphate buffer [pH 7.4], 1 mM MgCl_2_, 3 mM KCl, 150 mM NaCl, and 0.05% Tween-20). The data were analyzed using the MO.Affinity Analysis program version 2.2.4. and Graphpad Prism version 8.0.2.

#### NMR

Protein samples enriched with ^15^N were expressed in M9 minimal medium, containing 1 g/L of ^15^NH_4_Cl as the sole nitrogen source, and purified using the protocols described elsewhere.^20^ A sample of ^15^N-labeled CDNF at a concentration of 0.2 mM in 25 mM MES buffer (pH 7.0), 150 mM NaCl containing 10% D_2_O was used for the acquisition of 2D [^1^H,^15^N]-HSQC spectra. The α-synuclein was added to a sample with ^15^N-labeled CDNF, so that each protein achieved 0.2 mM final concentration. The recombinant monomeric human α-synuclein was produced in bacteria as described elsewhere.^56^

The NMR spectra were collected at 25°C in a Bruker Avance III 500 MHz spectrometer, equipped with a 5 mm z-gradient triple-resonance probe, located at the National Center for Structural Biology and Bioimaging (CENABIO/UFRJ). Spectra were processed and analyzed with the programs Topspin 1.3 (Bruker Corporation) and CARA 1.9.1.5, respectively. The chemical shifts used were obtained from the BioMagResBank database (CDNF: 19164; α-synuclein: 6968). The analyses and figures of the three-dimensional (3D) structures were performed with the Molmol 2K.2 software. The figures with the combined chemical shift difference were generated with the GraphPad Prism version 6.01.

#### α-synuclein aggregation assay by ThT

For kinetics experiments, the samples contained 10 μM ThT and 70 μM monomeric recombinant human α-synuclein alone^56^ or in combination with 70 μM recombinant human CDNF^20^ or 70 μM BSA (Merck, cat. no. 05470). These samples were incubated at 37°C in a 96-well plate (Costar, cat. no. 3631) together with Teflon spheres, 0.3 cm diameter. Every 5 min, the plates were shaken for 30 s, and fluorescence (excitation at 450 nm, emission at 477 nm) was monitored using a SpectraMax Paradigm Multi-Mode Microplate Reader. Each aggregation assay was performed in triplicate.

#### Bimolecular fluorescence complementation assay

HEK 293 cells were cultured at 37°C in a humidified incubator containing 5% CO_2_ in DMEM (Life Technologies-Invitrogen, Carlsbad, CA, USA) supplemented with 10% fetal bovine serum Gold (PAA, Colbe, Germany) and 1% penicillin/streptomycin (PAN, Aidenbach, Germany). The day before the transfection, HEK cells were seeded in 12-well plates (Costar, Corning, NY, USA) with coverslips (15 mm diameter) coated with gelatin (Merck, Darmstadt, Germany). Thirty minutes before the transfection, the cells were incubated in Opti-MEM (Life Technologies-Invitrogen, Carlsbad, CA, USA) and next transfected with equimolar amounts of the plasmids using Metafectene (Biotex, Munich, Germany) according to the manufacturer’s instructions for 48 h, similarly as described in Lazaro et al^25^. The constructs used were: VN aSyn (Addgene cat. no. 89470), aSyn VC (Addgene cat. no. 89471), VN CDNF (contains the N-terminal part of Venus fused to the human c-DNA for mature CDNF sequence inserted into VN aSyn vector via *Afl* II and *Xho* I sites), and CDNF VC (contains the human c-DNA for mature CDNF sequence inserted into VN aSyn vector via *Afl* II and *Xho* I sites, fused to the C-terminal part of Venus).

#### Immunocytochemistry

Forty-eight hours after transfection, the medium was removed and the cells were washed with PBS and fixed with 4% PFA for 30 min at RT. Cell permeabilization was performed with 0.5% Triton X-100 (Sigma-Aldrich, St. Louis, MO, USA) for 20 min at RT, followed by blocking in 3% BSA (Nzytech, Lisbon, Portugal) in PBS 1× for 1 h. Afterward, cells were incubated with primary antibodies: mouse anti-ASY (1:1,000, BD Transduction Laboratories, Franklin Lakes, NJ, USA) and rabbit anti-CDNF (1:1000, Sigma-Aldrich, St. Louis, MO, USA) overnight and secondary antibody Alexa Fluor 488 donkey anti-mouse IgG (Life Technologies-Invitrogen, Carlsbad, CA, USA) for 2 h at RT. Finally, cells were stained with DAPI (Carl Roth, Karlsruhe, Germany) (1:5,000 in PBS 1×) for 10 min, and the coverslips mounted on SuperFrost Microscope Slides treated with Mowiol (Calbiochem, San Diego, CA, USA), dried and stored at RT until further visualization and analysis. Images were acquired using an epifluorescence microscope (Leica DMI 6000B microscope, Leica, Wetzlar, Germany). For each condition, 20 images were taken with the same exposition time for each channel. Normalized image analysis was performed using Fiji^57^ open-source software using the region of interest (ROI) plugin and calculating the mean fluorescence intensity of each ROI.

#### Preparation of PFF

Mouse PFFs were prepared as previously described.^5^ Briefly, full-length monomeric mouse α-synuclein was expressed in *Escherichia coli*, purified, and then underwent shaking at 1,000 rpm for 7 days at 37°C so that a fibrillar structure was formed. PFFs were stored in aliquots at −80°C until use, when they were thawed at RT and sonicated in sterile PBS in Eppendorf tubes using a tip sonicator with 60 pulses at 0.5 s interval (Qsonica Microson XL 2000 Ultrasonic Liquid Processor, Newtown, CT, USA). For seeding of neuronal cultures, PFFs were sonicated at 0.1 mg/mL concentration and seeded at 2.5 μg/mL into culture medium. For *in vivo* mouse injections, PFFs were sonicated and injected at a concentration of 2.5 mg/mL and for rat injections at 2 mg/mL.

#### Primary neuronal culture and seeding of the α-synuclein PFFs

Neuronal cultures were prepared from embryonic day 16–17 (E16–17) mice (NMRI strain, Viikki Laboratory Animal Center, University of Helsinki) as described,^58^ with slight modifications. Briefly, the hippocampal or cortical areas were collected to Hank’s balance salt solution (HBSS, Thermo Fisher Scientific/Gibco, cat. no. 14170), washed with HBSS twice, dissociated with trypsin (0.1 mg/mL, 15 min at 37°C) in HBSS, treated shortly with DNase I (1 mg/mL, Roche) in HBSS containing 10% fetal bovine serum (Fisher Scientific/Gibco), and washed twice with HBSS + fetal bovine serum. To achieve single-cell suspension, the tissue was triturated with gentle pipetting in culture medium consisting of NeuroBasal medium (Thermo Fisher Scientific/Gibco) supplemented with B27 (Thermo Fisher Scientific/Gibco), 1% L-glutamine (Thermo Fisher Scientific/Gibco), and 0.2% Primocin (InvivoGen). The cells were plated on poly-D,L-ornithine-coated wells in culture medium on 96-well plates at a density of 25,000 cells per well, or on 24-well plates at 200,000 cells per well, or on 6-well plates at 1 million cells per well. Half of the volume of the culture media was replenished every 4–5 days.

The PFF seeding was performed as described.^5^ The primary hippocampal or cortical cultures at days *in vitro* (DIV) 7 were seeded with PFFs by replacing half of the medium in the wells with the culture medium containing the sonicated PFFs or the vehicle as control. Human recombinant CDNF, produced in CHO cells (Icosagen, Tartu, Estonia), was added to the cultures by replacing part of the medium, and PBS was used as the vehicle control. In the overexpression study, CDNF was expressed in cultured neurons through transduction at DIV 2 with a lentiviral vector (LV) carrying CDNF under human *Synapsin1* (hSYN) promoter, and lentiviral overexpression of EGFP (enhanced green fluorescent protein) was used as a control.

#### Construction of transfer plasmids and production of lentiviral vectors

A 452 nt fragment of human SYN1 gene promoter was PCR amplified using Phusion high-fidelity DNA polymerase (Thermo Scientific) and the following primers: *Bcu*I_hSYN_for 5′- ATACTAGTAGTGCAAGTGGGTTTTAGGACC-3′ and EcoRI_hSYN_rev 5′- TGGAATTCGACTGCGCTCTCAGG-3′, digested with FastDigest-*Bcu*I/-EcoRI (Thermo Scientific) and cloned into the pCDH-CMV-MSC-T2A-EGFP vector (System Biosciences) digested with the same restriction enzymes, resulting in pCDH-hSYN-MSC-T2A-EGFP construct. To obtain the pCDH-hSYN-hCDNF lentivirus transfer plasmid, coding sequences of hCDNF (with original N-terminal signal sequence) were PCR amplified from human tissue cDNA with the following primers: CDNF_For 5′-TTGGATCCATGTGGTGCGCGAGCC-3′, CDNF_Rev GAGTCGACTAGAGCTCTGTTTTGGGGTGTG-3′, digested with FastDigest-BamHI/-SalI (Thermo Scientific) and cloned into a pCDH-hSYN-MSC-T2A-EGFP vector digested with the same enzymes. All plasmid constructs were verified by DNA sequencing. Lentiviral vectors were produced as described.^59^

#### Sequential extraction of proteins for western blot

To harvest the soluble and the insoluble proteins into separate fractions, we first used the milder non-ionic detergent Triton X-100 and next the strong anionic detergent SDS for sequential protein extraction.^5^ The neuronal cultures on 6-well plates were placed on ice, rinsed twice with PBS, and then scraped into suspension in ice-cold TX-lysis buffer (1% Triton X-100 in TBS containing complete Mini protease inhibitor cocktail and PhosSTOP phosphatase inhibitor cocktail [Roche]) and sonicated 10 times with a 0.5 s pulse, at 10% power (Qsonica Microson XL 2000 Ultrasonic Liquid Processor, Newtown, CT, USA). After 30 min incubation on ice, the samples were centrifuged at 100,000 × *g* at 4°C for 30 min. The supernatants (i.e., TX-extracts) of each sample were collected, and a small aliquot of each was saved for protein concentration measurements (DC Protein Assay, Bio-Rad Laboratories, Hercules, CA, USA). The remaining pellets were washed by adding 250 μL of ice-cold TX-lysis buffer, repeating the sonication and centrifugation steps, and then discarding the supernatants. Next, 125 μL of SDS-lysis buffer (2% SDS in TBS with protease and phosphatase inhibitors) was added to the pellets, the samples were tip-sonicated, the SDS extracts were collected, and small aliquots were saved for protein concentration measurements. At the end, 5× Laemmli buffer was added to the Triton X-100 and SDS samples and they were stored at −20°C for western blot analysis.

#### Western blotting

The samples were heated at 95°C for 5 min, loaded on 12% polyacrylamide gels, and subjected to SDS-PAGE. After electrophoresis, the proteins were transferred to a nitrocellulose membrane. The membrane was fixed with 4% PFA and 0.01% glutaraldehyde in PBS for 30 min to retain α-synuclein.^60^ After fixation, the membrane was washed 3 × 5 min in TBS. The membrane was then blocked in 3% milk in TBS-T (0.01% Tween-20 in TBS) for 60 min and then incubated with primary antibody (in TBS-T with 3% skim milk) overnight at 4°C. Next day, the membrane was washed 3 × 15 min in TBS-T, incubated with secondary antibody (in TBS-T with 3% skim milk) for 1 h at RT, and then washed 3 × 15 min with TBS-T and once for 15 min with TBS. The secondary antibodies used were IRDye from LI-COR Biosciences (Lincoln, NE, USA) at 1:10,000 dilution. The membranes were scanned on an Odyssey CLx imaging system from LI-COR Biosciences, and the band intensities were analyzed from the images using the Odyssey CLx software. Antibody used to detect total α-synuclein was the ab1903 (Abcam) and for detection of the pSer129 α-synuclein, the ab51253 (Abcam).

#### Immunofluorescent staining of hippocampal cultures

At the end of the experiment, the cells were fixed with 4% PFA in PBS for 20 min at RT and then washed thrice with PBS and permeabilized in PBS-T (0.2% Triton X-100 in PBS) for 15 min at RT. PBS-T was then replaced with blocking solution (5% normal horse serum in PBS-T) and incubated for 1 h at RT. The blocking serum was replaced with primary antibodies diluted in blocking serum (anti-pSer129 α-synuclein 1:2,000 dilution, ab51253, Abcam, and anti-NeuN 1:400 dilution, MAB377, Millipore) and incubated overnight at 4°C, under slight rotation. Next day, the cells were washed with PBS thrice (10 min/wash) and incubated with the fluorescent secondary antibodies diluted in PBS-T (goat anti-rabbit AlexaFluor568 and donkey anti-mouse AlexaFluor647, Life Technologies) for 1 h, after which the cells were washed thrice with PBS. Last, DAPI (Invitrogen, stock 1 mg/mL; diluted to 1:5,000 in PBS) was added to each well for 15 min incubation at RT; the cells were washed thrice with 1× PBS and left in PBS at 4°C until fluorescence scanning.

#### Neuronal culture scanning and image analysis

The cells on 96-well plates were scanned using CellInsight CX5 High Content scanner (Thermo Fisher Scientific) with 10× objective. Multichannel images (NeuN, pSer129 α-synuclein and DAPI) were acquired from 16 fields per well and quantification of NeuN-positive cells (i.e., neurons) with or without pSer129 α-synuclein-positive soma inclusions was performed with CellProfiler and CellAnalyst software packages.^61^, ^62^, ^63^ Briefly, these programs were used to classify the image pool and obtain the total number of NeuN-positive cells and the number of NeuN-positive cells containing large intra-somal pSer129 α-synuclein inclusions.

#### Internalization of radiolabeled PFFs

Mouse α-synuclein PFFs were radiolabeled with radioactive iodine (^125^I) to allow characterization of their internalization to hippocampal cultures. Sonicated PFFs were iodinated using the lactoperoxidase method.^64^ Hippocampal cultures (DIV7) on 24-well plates were placed on ice, and the iodinated α-synuclein PPFs, mixed with different concentrations of CDNF, persephin, or vehicle, were added to the culture. The plates were moved to a 37°C water bath for 60 min to allow internalization. After this, the plates were washed 3× with PBS and 5 min with acid buffer comprising 0.2 M acetic acid and 0.5 M NaCl (pH 2.8) to remove proteins bound to the cell surface. The cells were scraped into Eppendorf tubes and centrifuged for 3 min at 3,000 rpm in a table-top centrifuge. The acid buffer was removed, and 1 M NaOH was added to lyse the cells. Radioactivity of the lysates was quantified on a Wallac/LKB Wizard 1470 gamma counter.

### In vivo

#### *In situ* PLA experiment

For the PLA experiment, 4 rats were given a single injection of human CDNF to the right striatum in stereotaxic surgery, total 10 μg of CDNF in 4 μL as described below. The coordinates relative to bregma were A/P +1.0, M/L −2.7, D/V −5.5, and the flow rate was 0.5 μL/min. The rats were perfused 2 h later as described below.

To measure the *in vivo* interaction of α-synuclein and CDNF, paraffin sections on slides were processed as below for deparaffinization, antigen retrieval, and washing. A Duolink PLA (Sigma-Aldrich, cat. no. DUO92101-1KT) kit was used according to the manufacturer’s protocol, all steps performed at RT unless otherwise specified. Slides were then coverslipped with Duolink *In Situ* Mounting Media with DAPI. A reaction with primary antibodies for DAT and α-synuclein (4D6) was used as a positive control,^27^ and for the negative control sections were not incubated in any primary antibody, but otherwise the protocol was followed.

#### Animals

Young male mice (2–3 months old at start of experiment, C57BL/6JRccHsd), aged male mice (11 months old at start of experiment, C57BL/6JRj), young male rats (Hsd:HanWistar and Sprague-Dawley), or aged male rats (13 months old at start of experiment, Hsd:HanWistar) were used. All animals were under a 12-h light/dark cycle and had access to *ad libitum* food and water. Mice were housed separately for the duration of the experiment, while rats were housed in groups of two. Experiment durations were from 3 to 6 months. All surgeries and behavioral assays were carried out at the University of Helsinki animal laboratory facilities. All animal experiments were approved by the Finnish National Board of Animal Experiments and were carried out according to the European Community guidelines for the use of experimental animals; license number ESAVI/7812/04.10.07/2015. All guidelines for reporting the use of the animals were followed, and 3R principles were adhered to. Sample sizes were chosen based on number of treatment groups and previous experience with stereotaxic injection experiments. Animals were separated into treatment groups based on rotarod behavior analysis.

#### PFF injections

Stereotaxic injections for the animals were performed under isoflurane anesthesia (mice: 3.5% induction, 1.5%–2% maintenance; rats: 4.5% induction, 2%–3% maintenance). Animals were placed in a stereotaxic frame (Stoelting, Wood Dale, IL, USA), lidocaine (Orion Pharma, Finland, >0.1 mL) was applied under the skin on top of the head to anesthetize the area and stem bleeding, and a small incision was made to expose the skull. Burr holes in the skull were made unilaterally with a micro drill. A 33G steel needle with a 10 μL syringe (Nanofil, World Precision Instruments) was used to inject the PFFs in all experiments. For mice, 2.5 μL was injected into the striatum at a 10° angle (coordinates from bregma: A/P 0.7, M/L −2.2, D/V −3.0 from the skull), with a flow rate of 0.1 μL/min and letting the needle rest 5 min after the injection finished. For rats, 2 μL was injected into 2 sites (total volume 4 μL) of the striatum at a 10° angle (coordinates from bregma: A/P +1.6, M/L −2.8, D/V −6.2; A/P 0.0, M/L −4.1, D/V −6.2 from the skull), using a flow rate of 0.5 μL/min and letting the needle rest for 5 min after each injection. After the surgery, all animals received carprofen for pain relief (Rimadyl, Pfizer, subcutaneous [s.c.] 5 mg/kg). Animals were placed in a separate recovery cage until they awakened, and then they were returned to their home cage.

#### CDNF administration

For single injection of human CDNF to mice and rats, stereotaxic injections were performed as above. Recombinant human CDNF was injected into the same location in the striatum as the PFFs for mice. CDNF (stock concentration 9.6 μg/μL, Biovian, Turku, Finland) was diluted in sterile PBS to 2.5 μg/μL and injected in 4 μL, with a flow rate of 0.5 μL/min, and the needle was let to rest for 5 min after the injection. PBS was used as a vehicle control.

For chronic infusion, CDNF was diluted from the stock solution (concentration 9.6 μg/μL, Biovian, Turku, Finland) using sterile PBS to correspond to doses of 1.5 μg/24 h or 3 μg/24 h, and osmotic minipumps (model no. 2004, 0.25 μL/h, 4-week duration, Alzet, Cupertino, CA) were filled under a fume hood in aseptic conditions to avoid bacterial contamination. For vehicle, sterile PBS (pH 7.4) was used. The pumps were weighed before filling, after filling, and after removal. The pumps were filled the day before implantation and placed into a tube containing saline and then into a 37°C incubator overnight in order to acclimate them. The rats were anesthetized using isoflurane, put into the stereotaxic frame, the skull was exposed, and a burr hole was drilled as described above. The brain cannula (Brain Infusion Kit 2, 28G, 3–5 mm penetration, Alzet, Cupertino, CA) was placed into the striatum (coordinates from bregma: A/P +1.0; M/L −2.8; D/V −5.0). This was then secured to the skull using three stainless steel screws and polycarboxylate cement (Aqualox, VOCO, Germany). The tubing and the minipump were placed subcutaneously between the shoulders of the rats. The rats were given carprofen (Rimadyl, Pfizer, s.c. 5 mg/kg) and buprenorphine for pain relief immediately after the surgery and the following day.

#### Cylinder test

Animals were placed inside a clear, plexiglass cylinder elevated on a clear platform with a video camera underneath that was attached to a computer to record. Mice were recorded for 5 min and rats for 10 min, and the videos were viewed and analyzed by an experimenter blinded to the treatments. Touches to the wall of the cylinder by ipsilateral paw alone, contralateral paw alone, or both paws were counted. A naive animal is expected to use both left and right paws approximately 50% of the time. All animals had at least 20 touches on the cylinder wall.

#### Rotarod

Mice were placed on a Rotarod device (Ugo Basile) on a rotating rod accelerating from 4 rpm to 40 rpm in 240 s. For training, mice had one session where they were placed in this accelerating paradigm until they fell off. Then a few hours later they were tested, and the time they were able to stay on the rod was recorded. For rats, training and testing were the same, except that the accelerating rod was set to 4–40 rpm in 300 s.

#### Coat hanger test

A coat hanger was affixed to a metal rod and placed on a table so that the bottom of the coat hanger was 30 cm from the tabletop. The mouse was removed from the home cage and allowed to grip the center of the bottom of the coat hanger with its forepaws. Once the mouse had a secure grip, it was released, and the time was recorded until the mouse fell or to a maximum of 60 s.

#### Tissue collection

For collection of the whole brain for immunostaining in young mice, the rodents were anesthetized with a high dose of sodium pentobarbital (90 mg/kg intraperitoneally [i.p.], Orion Pharma, Finland) and perfused transcardially with PBS and then 4% PFA. For free-floating sections, the brains were removed, placed in 4% PFA overnight, then later in 20% sucrose, and stored at 4°C. Brains were frozen in a cryostat (Leica CM3050) and cut into 30 μm sections, placed in PBS, and later transferred to cryopreservant solution (20% glycerol, 2% DMSO in PBS) and stored at −20°C. Striatum and *substantia nigra* were collected for immunostaining. For paraffin sections, brains were put into 4% PFA for a few days (with changes to fresh 4% PFA regularly), then embedded in paraffin. Sections were cut on a microtome into 10 μm thickness and stored at 4°C for further processing.

#### Immunostaining of sections

For immunostaining of free-floating sections, the sections were thawed at RT and PBS was used for rinsing. Sections were blocked using hydrogen peroxide (0.3% H_2_O_2_ in PBS) for 30 min to quench endogenous peroxidase activity and blocked with 4% BSA and 0.3% Triton X-100 in PBS for 1 h. Sections were then incubated with the primary antibody in BSA blocking buffer overnight at 4°C. After incubation with the primary antibody, sections were rinsed with PBS and incubated with the secondary antibody (Vector Laboratories anti-mouse or anti-rabbit biotinylated secondary antibody cat. no. PK-4002 or PK-4001, dilution 1:200) in BSA blocking buffer for 1 h at RT. Then sections were incubated with avidin-biotinylated horseradish peroxidase (ABC Kit, Vector Laboratories) in PBS for 1 h and developed with 0.05% 3,3-diaminobenzidine-4 HCL (DAB) (DAB peroxidase substrate kit, SK-4100, Vector Laboratories) in water for 30–60 s, rinsed with PBS, and placed on slides. For controls, either the primary or the secondary antibody was omitted. Slides were allowed to dry overnight at RT, dehydrated, and mounted with Coverquick 2000 (Q PATH) mounting medium. The experimenter was blind to the treatment groups throughout the process.

For DAB staining of paraffin sections, after deparaffinization, sections underwent antigen retrieval by heating them up in citraconic anhydride (0.05% citraconic anhydride in Milli-Q water [pH 7.4]) without boiling. Slides were then cooled, and endogenous peroxidase activity was quenched using 3% H_2_O_2_ in TBS for 30 min at RT. Slides were washed with TBS, and then sections were encircled with a PAP pen, washed with TBS-T, and blocked with 1.5% normal horse serum (Vector) in TBS-T for approximately 20 min. The primary antibody was then added onto the sections and the slides were incubated at 4°C overnight. The next day, the sections were incubated in the secondary antibody solution (horse-anti-rabbit 1:200, Vector) for 1 h at RT and then in the avidin-biotinylated horseradish peroxidase complex solution (ABC kit, Vector) for 30 min. Slides were rinsed with TBS-T, and then the sections were incubated with DAB for 5 min, rinsed with TBS, dehydrated, mounted, and coverslipped with Coverquick 2000 (Q PATH) mounting medium. For fluorescent staining of paraffin sections, the same steps were followed as above but without quenching with hydrogen peroxide, the avidin-biotin reaction, or dehydration. Slides were then washed with TBS-T, briefly with Milli-Q water, coverslipped with Vectashield HardSet mounting medium with DAPI (Vector, H-1500), and after drying briefly at RT stored at 4°C.

#### Optical density

TH-positive fiber density in the striata of 3-month-old mice was determined using immunostained coronal striatal sections at approximately A/P +1.32 mm, 0.7 mm, and 0.35 mm relative to bregma. Immunostained sections were scanned with a Pannoramic 250 Flash II scanner (3DHISTECH, Budapest, Hungary) at the service provided at the Institute of Biotechnology, University of Helsinki. Images were taken with the CaseViewer (3DHISTECH) software and analyzed using Image-Pro Analyzer 7.0 (Media Cybernetics). The contralateral side of each striatal section was used as a control, the corpus callosum was used to eliminate unspecific background staining, and all analyses were done by the experimenter who was blind to the treatments.

#### Counting of pSer129-positive inclusions

Immunostained sections were scanned with Panoramic 250 Flash II scanner (3DHISTECH, Budapest, Hungary) at the service provided in the Institute of Biotechnology, University of Helsinki. CaseViewer (3DHISTECH) software was used to analyze the sections. The slides were scanned with single-layer 20× magnification allowing reliable manual identification of pSer129-positive inclusions in the *substantia nigra* by an experimenter trained to recognize pSer129-positive staining and blinded to the treatments. 5 to 19 sections from each brain were used from approximately A/P −4.5 to 6.0 relative to bregma. Every positive inclusion (not differentiating whether it was intracellular, extracellular, or in a neurite) was manually marked using the annotation function on each section, and the results were calculated as the average number of pSer129 inclusions per section.

#### Confocal microscopy

LSM 700 (Carl Zeiss) confocal microscope, LCI Plan-Neofluar 63×/1.30 glycerol immersion objective at RT, and Zen Black acquisition software (Carl Zeiss) were used for confocal microscopy image acquisition. A combination of Zen Blue Lite (Carl Zeiss), and CorelDraw programs were used for image processing.

#### Statistical analysis

Statistical analyses were performed with GraphPad Prism 7. To compare data from different culture plates and independent experiment repeats, the data were normalized to the control groups. The statistical significance was estimated using one-way ANOVA or two-tailed Student’s t test, where p < 0.05 was considered significant. Where appropriate, the data were subjected to post hoc Dunnett’s, Bonferroni, or Tukey’s test. The values are given as mean ± SEM. Tests that ensure normal distribution of data and equal variances were performed before other statistical tests were used.

## Supplemental information

Document S1. Figures S1–S9 and Tables S1 and S2

Document S2. Article plus supplemental information
