# Supplementary material for: Cerebral dopamine neurotrophic factor reduces α-synuclein aggregation and propagation and alleviates behavioral alterations in vivo
Source: Mol Ther. 2021 May 1;29(9):2821–40. doi: 10.1016/j.ymthe.2021.04.035 (PMC8417450; doi:10.1016/j.ymthe.2021.04.035)
Supplement: Document S1. Figures S1–S9 and Tables S1 and S2 [file mmc1.pdf]

YMTHE, Volume *29*

Supplemental Information

Cerebral dopamine neurotrophic factor reduces α-synuclein aggregation and propagation and alleviates behavioral alterations *in vivo*

Katrina Albert, Diana P. Raymundo, Anne Panhelainen, Ave Eesmaa, Liana Shvachiy, Gabriela R. Araújo, Piotr Chmielarz, Xu Yan, Aastha Singh, Yraima Cordeiro, Fernando L. Palhano, Debora Foguel, Kelvin C. Luk, Andrii Domanskyi, Merja H. Voutilainen, Henri J. Huttunen, Tiago F. Outeiro, Mart Saarma, Marcius S. Almeida, and Mikko Airavaara

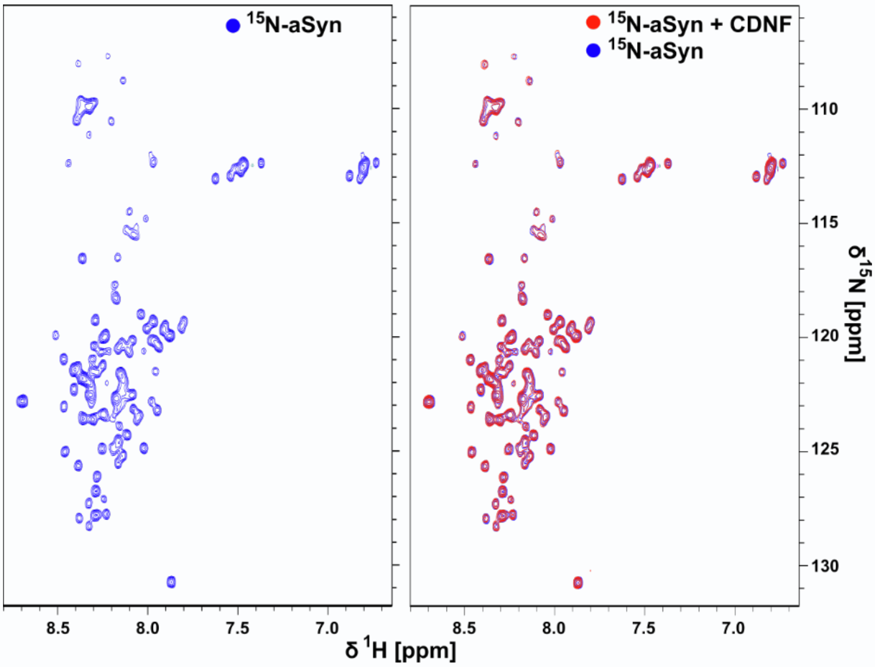

Figure S1. Effect of CDNF on ^{15}N-α-synuclein. 2D [^{1}H-^{15}N]-HSQC spectra of the ^{15}N-labeled human recombinant α-synuclein in the absence (blue) and in the presence (red) of CDNF. Spectra were collected at 25 °C with 0.15 mM of each protein.

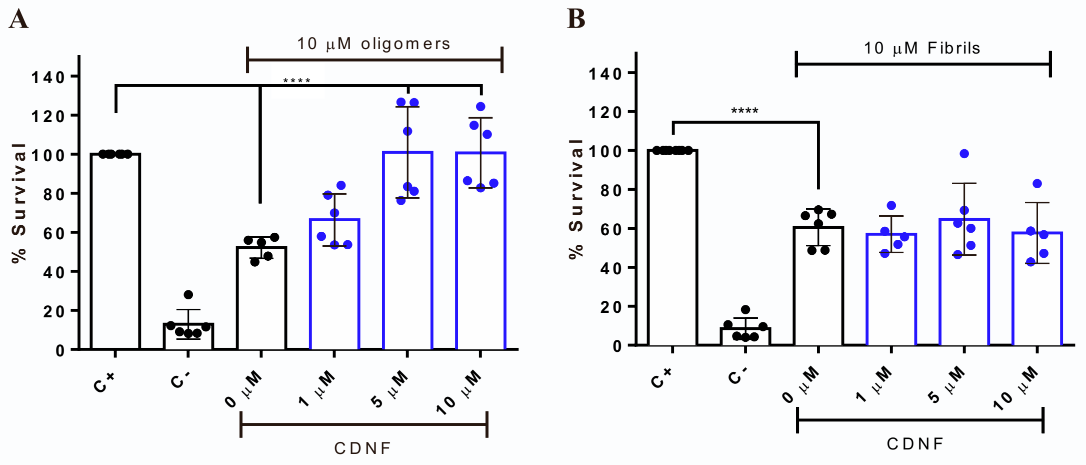

Figure S2. Cell viability assay by MTT in cells challenged with aSyn (n = 3). H4 cells were cultured for 16 hours and then pre-treated with CDNF or buffer for 1 hour prior to the addition of 10 µM oligomeric or fibrillar aSyn. After 24 hours, cell viability was visualized using MTT assay. The positive control (C+) corresponds to cells without treatment whereas the negative control (C-), cells treated with 0.1% Triton X-100. Error bars represent the standard deviation. One-way ANOVA **** $P < 0.0001$.

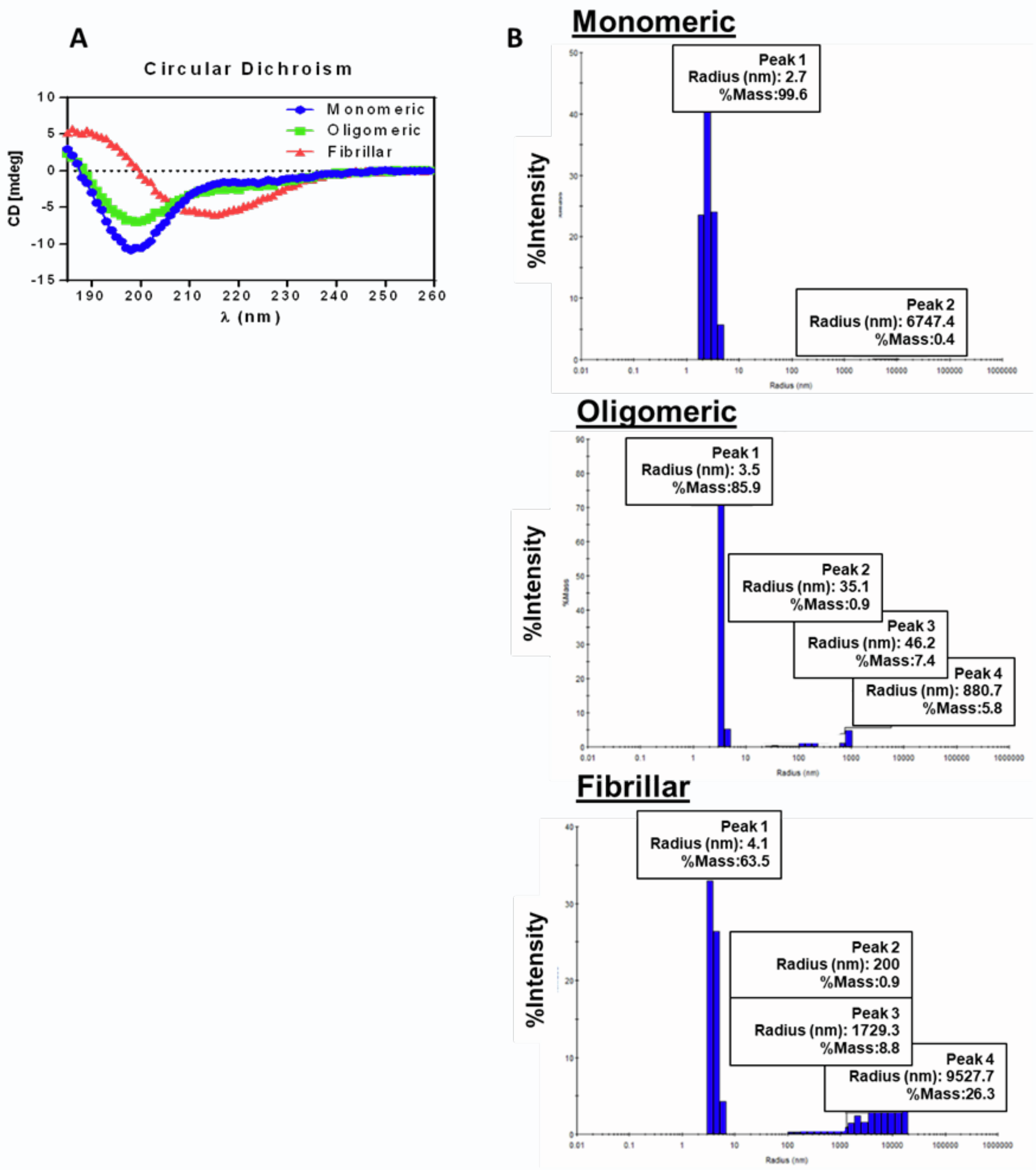

Figure S3. Biophysical characterization of α-synuclein aggregation. 140 µM of monomeric α-syn protein was incubated at 37 °C, 800 rpm for 48 hours or 10 days to achieve oligomers and fibrils, respectively. Both oligomers and fibrils were submitted to Circular Dichroism spectroscopy (A) and Dynamic Light Scattering (B). As shown in panel A, monomeric synuclein has a typical intrinsically disordered spectrum (line in blue) with negative band near 195 nm, similar to the oligomeric α-syn spectrum (green line). The fibrils (red line) present the characteristic spectrum with negative band at 218 nm and positive band at 195 nm, indicative of well-defined antiparallel β-pleated sheets (β-helices). As shown in panel B the monomeric preparation has 99.6% of purity with 2.7 nm of radius. The oligomeric and fibrillar preparation presents different populations with 3.5 nm up to 9,527.7 nm of radius.

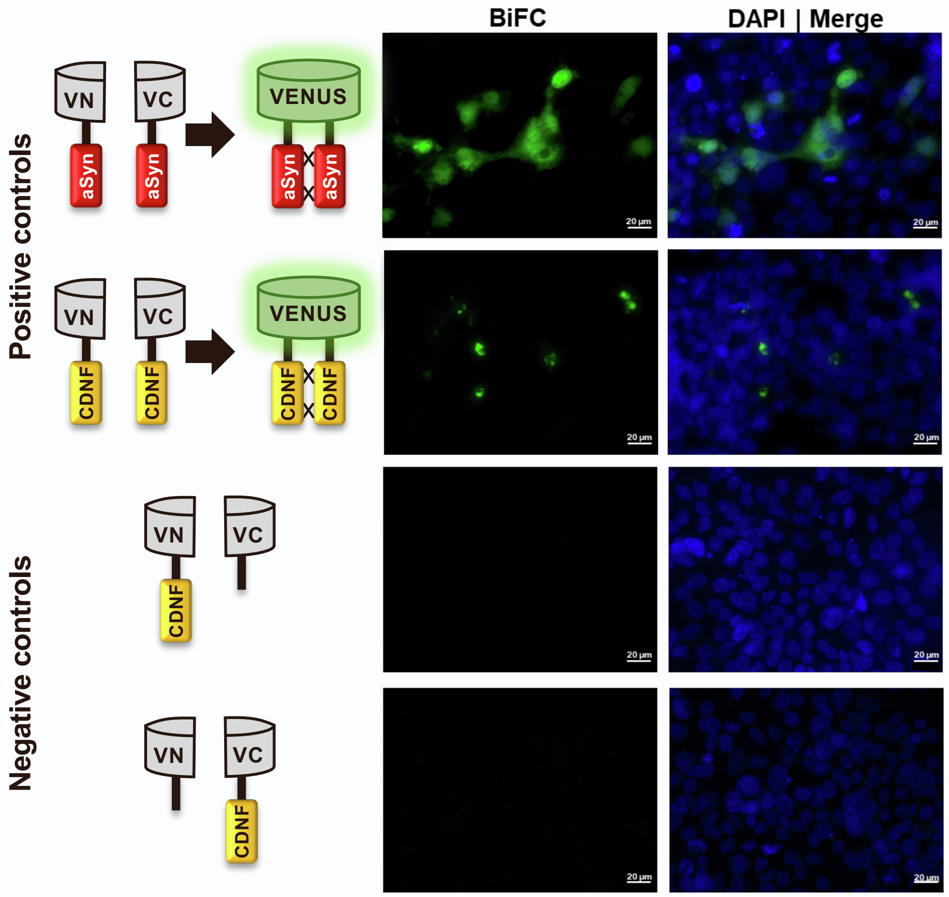

Figure S4. Controls used for the BiFC studies. VN-aSyn and aSyn-VC, and VN-CDNF and CDNF-VC combinations were used as positive controls. Negative controls were obtained by transfecting HEK cells with VN-CDNF and VC, or VN and CDNF-VC constructs. Cells were imaged using a LEICA epifluorescence microscope. BiFC interactions are presented in green and DAPI staining in blue

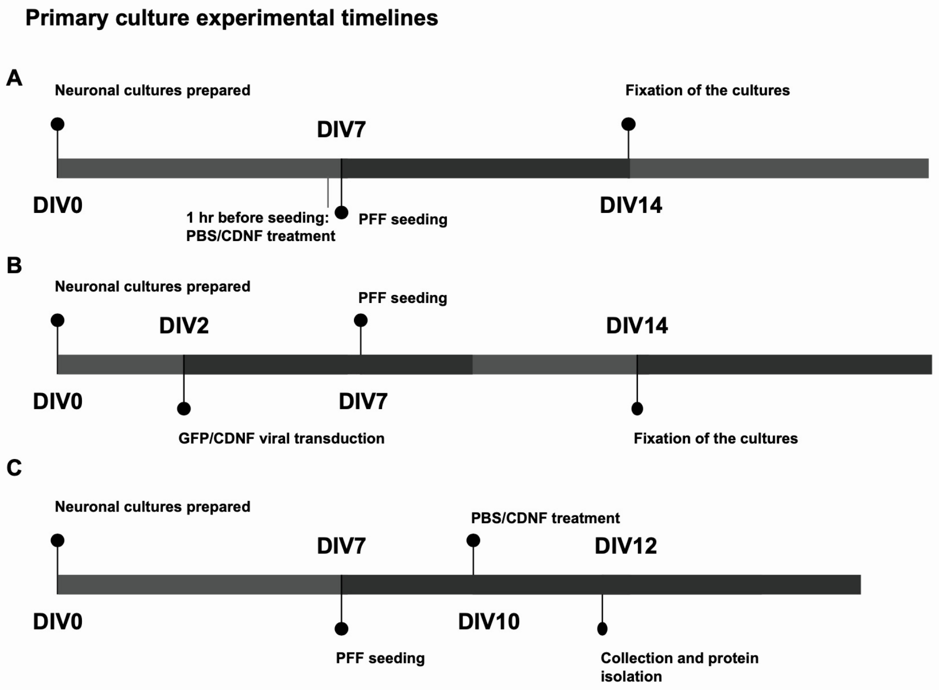

Figure S5. Timelines of in vitro neuronal culture experiments. (A) Timeline of PFF application and CDNF treatment, corresponds to Figure 5B. (B) Timeline of PFF application and CDNF viral transduction, corresponds to Figure 5C. (C) Timeline of PFF seeding, CDNF treatment, and protein isolation, corresponds to Figure 5D-E

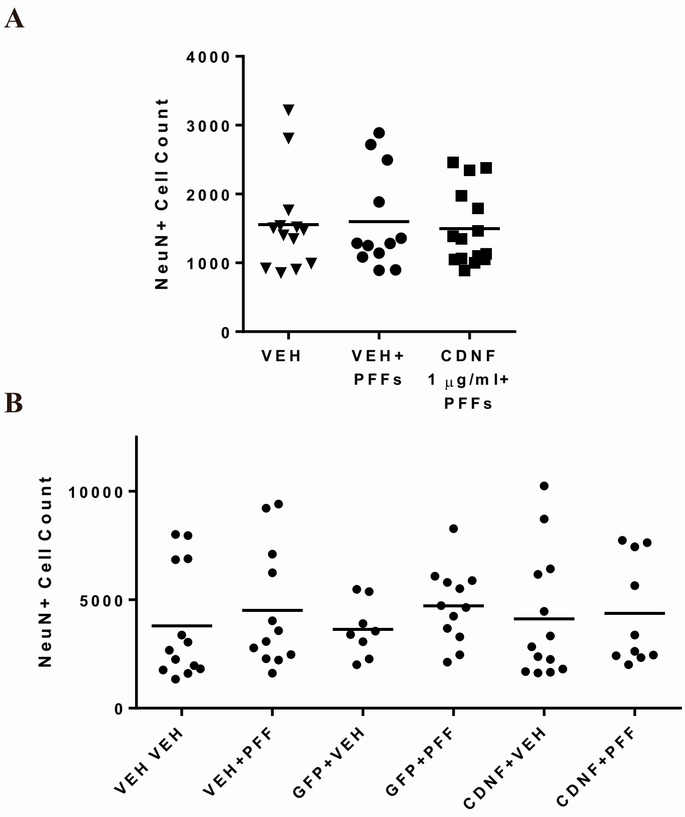

Figure S6. PFF-seeding or any of the other treatments did not affect the survival of neuronal cultures. (A) Number of NeuN-positive cells in VEH- or PFF-seeded cultures and the effect of CDNF pretreatment on them. (B) Number of NeuN-positive cells in non-transfected and non-seeded or seeded (VEH VEH and VEH+PFF) or in cultures transfected with lentiviral vector carrying GFP or CDNF for overexpression and seeded with PFFs.

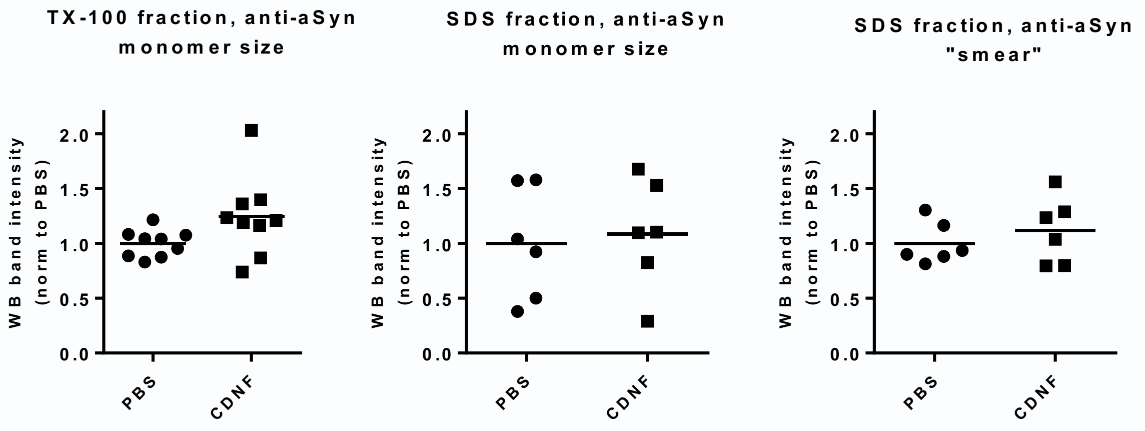

Figure S7. PFFs were seeded (2.5 ug/ml) to primary cortical cultures at DIV7 and CDNF (125 ng/ml) was added at DIV10 and sequential extraction of proteins was done at DIV12. CDNF treatment did not affect the level of aSyn monomer in TX100 nor SDS fractions, and did not affect the intensity of the smears appearing in SDS fractions.

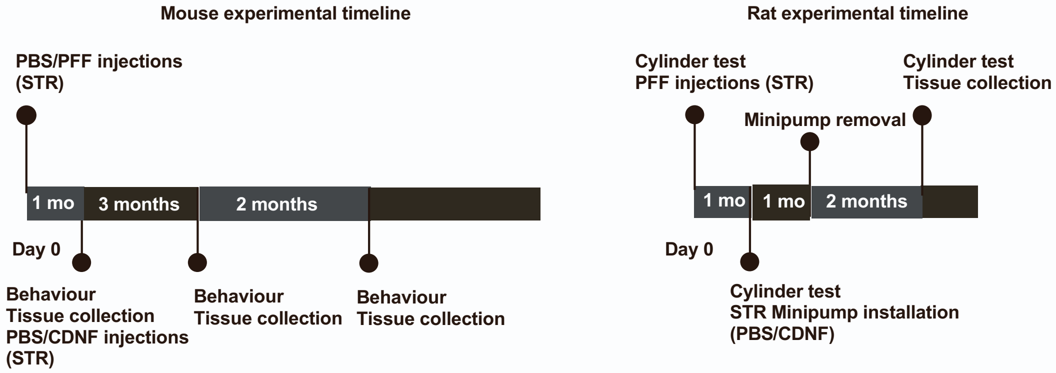

Figure S8. Timelines of animal experiments. For the experiments with mice, PFFs were injected to the striatum (STR) of 3-month and 11-month old mice at day 0 and 1 month later vehicle (PBS) or CDNF was injected into the same location in the STR. Behavioral tests (cylinder test, rotarod, and coat hanger test) were performed at 1, 3, and 6 months. Animals were euthanized at 6 months post-PFFs and the tissue was collected for immunohistochemistry. For the experiments with rats, PFFs were injected at day 0 to the STR of 1-year old rats. Minipumps with vehicle (PBS) or CDNF at two different doses (1.5 µg/day or 3 µg/day for 30 days) were installed either 1 month after PFF injection. After 1 month, the minipumps were removed. Cylinder test was performed at baseline (before fibril injections), at 1 month (before minipump installation), and at 4 months (3 months after minipump installation). Tissue was collected 4 months after fibril injection for immunohistochemistry.

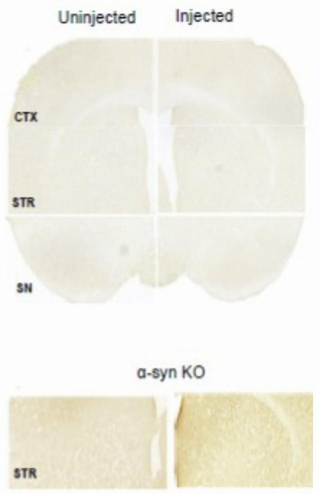

Figure S9. Controls for in vivo studies of pSer129 propagation. (A) Wild-type mouse injected with PBS at 6 months and immunostained with pSer129 at 6 months post-injection. No visible staining was present, 2.5x magnification. (B) aSyn knock-out mouse injected with PFFs and immunostained with PSer129 at 1 month post-injection. No visible staining was present, 10x magnification.

Table S1. List of primary antibodies used.

Antigen	Species	Manufacturer	Catalogue number	Dilution
Tyrosine hydroxylase (rat sections)	mouse	Chemicon	MAB318	1:2,000
Tyrosine hydroxylase (mouse sections)	rabbit	Chemicon	MAB152	1:500
α-synuclein pSer129	rabbit	Abcam	ab51253	1:10,000
α-synuclein [4D6]	mouse	Abcam	ab1903	1:500
Dopamine transporter (DAT)	rabbit	Merck-Millipore	AB1591P	1:250
Human CDNF	rabbit	Icosagen	N/A	1:1,000
Human α-synuclein	mouse	BD	610787	1:1,000
Human CDNF	rabbit	Sigma	PRS4343	1:1,000
DAPI	-	Carl Roth	6335.1	1:5,000

Table S2. Solvent accessibility of CDNF amino acids with δΔ> 0.03 ppm upon addition of α-synuclein.

Amino acids	Solvent accessibility (%)	Area exposed (A^2)	Physic-chemical properties
**LEU18**	**0.1**	**0.3**	**Hydrophobic**
**ASN19**	**15.0**	**41.9**	**Polar Neutral**
**ARG20**	**26.2**	**98.5**	**Positive**
**TYR22**	**5.4**	**19.2**	**Aromatic**
**LYS23**	**33.1**	**110.1**	**Positive**
**LEU25**	**0.5**	**1.4**	**Hydrophobic**
**ILE26**	**36.8**	**111.0**	**Hydrophobic**
**VAL30**	**7.9**	**21.7**	**Hydrophobic**
**PHE32**	**21.7**	**74.8**	**Aromatic**
TYR58	11.1	39.8	Aromatic
**SER78**	**12.3**	**29.4**	**Polar Neutral**
MET81	16.1	49.3	Apolar Neutral
**ALA83**	**0.6**	**1.4**	**Apolar**
VAL118	14.9	40.8	Hydrophobic
GLN123	38.7	116.8	Polar Neutral
SER127	32.0	76.4	Polar Neutral
